# A mathematical perspective on Romanisation: Modelling the Roman road activation process in ancient Tunisia

**DOI:** 10.1371/journal.pone.0309752

**Published:** 2024-09-25

**Authors:** Nataša Djurdjevac Conrad, Robin Chemnitz, Margarita Kostré, Fleur Schweigart, Friederike Fless, Christof Schütte, Benjamin Ducke

**Affiliations:** 1 Zuse Institute Berlin, Berlin, Germany; 2 Deutsches Archäologisches Institut, Berlin, Germany; 3 Freie Universität Berlin, Institut für Mathematik und Informatik, Berlin, Germany; Ariel University, ISRAEL

## Abstract

Romanisation is a multi-faceted historical phenomenon with profound and lasting cultural impact on the ancient world. In the modern-day territory of Tunisia, this is particularly manifest during the first four centuries AD, under the reign of the Roman Empire. We derive a reduced, operational concept of Romanisation as a cultural diffusion process that is observable in the archaeological remains of the Roman era settlement system. We then introduce a novel mathematical model that computes spatio-temporal approximations for the Romanisation of the settlement system. The model is based on the concept of temporal road activation and makes minimal assumptions regarding input data quality. The results of our study contribute to the understanding of the time dynamics of the region’s road network, under the influence of Romanisation. Our model can be applied in similar archaeological research scenarios, to generate spatio-temporal backbones for the analysis of otherwise intractably complex social processes.

## 1 Introduction: Romanisation as cultural diffusion

The ancient Romans are a striking example of a civilisation that succeeded in spreading its distinctive cultural traits across a vast spatial domain, creating what might be considered history’s first global power [1–4]. These traits included architecture, crafts and artistic styles, technology and industry, engineering and trade organisation, civil and military administration, land use and surveying, writing and education [5–8]. In the following, we introduce a new mathematical model for exploring selected aspects of *Romanisation*, the multi-faceted phenomenon of cultural transformation that occurred across the vast and diverse dominion of the Roman Republic and its successor, the Roman Empire ([9]). We have chosen modern-day Tunisia, the heartland of the ancient Roman province of Africa Proconsularis, as an application case study for our model, since it provides a particularly illustrative example of Romanisation of material culture. In this part of the western Mediterranean world, the Romans long fought and finally subdued an advanced and powerful Phoenician-Punic (Carthaginian: [10]) civilisation [4, chapter 1], [11]. It then reshaped urban (the rebuilding of Carthage as a Roman city being the most famous example: [12]) and rural life with unprecedented comprehension, leaving behind a spectacularly rich archaeological record of the first four centuries AD (for an overview: [13]; for a similar perspective from another Roman province: [14]).

Romanisation is defined in this article as a complex process of cultural diffusion. In doing so, we are aware that the use of the term ‘Romanisation’ is associated with a controversial discussion in archaeology and history ([15] chapter 2). This debate is, among other things, about the evaluation of the actors’ behaviour. The actual significance of Romanisation to contemporaneous societies has also been challenged, e.g. by pointing out its ‘convenient’ compatibility with the colonial mentalities of more recent times [15]. But regardless of underlying social complexities or changing interpretative trends, the material manifestations of Roman influence are universally identifiable in the archaeological record [16].

Some of the associated cultural change may have been caused by willing adoption of the many attractive traits of Roman culture and state organisation [17], while others might have met with resistance [18–20]. But the Roman conquerors also never hesitated to enforce change, be it in the political, economical, spiritual or military spheres [21]. Eventually, every region of the Roman Empire developed its unique blend of indigenous and foreign features. This is quite visible in the rich Roman heritage of this study’s geographical focus area, modern day Tunisia (perhaps most impressively represented by Tunisia’s famous Roman mosaics: [22]). Here, Romanisation was most effective in the spheres of municipal administration and urbanisation, with the Roman Empire undertaking a most comprehensive reshaping of settlement system, land use and architecture (see [23, 24]). This, however, was built on strong pre-Roman roots, some of which remained visible centuries after their incorporation into the Roman domain.

We further presume that, in order for any cultural trait to spread, it has to be transported across a distance in space by the ‘sender’ and then successfully transmitted to the ‘receiver’. There is a crude analogy here to the concepts of *susceptibility* and *infection*, and we will make use of this to transfer some basic mechanics from well-established epidemiological models. We assume this to be a valid approach, since disease spread has an essential social component (a virus cannot travel far by itself). Crucially, however, we do not adopt the view that cultural diffusion, as a whole, is equivalent to the infection of organisms in either process or pattern.

By extended analogy, a political entity will be most successful in spreading its traits, thereby extending its control, if it is capable of two things: Building the infrastructure needed for social interaction across space (see e.g. [25–28]), and providing the incentives that benefit the acceptance of its rules and ideas. The ancient Romans are particularly famous for their road-building capabilities (this has long been acknowledged by scholars: [29–31]) and integrative political skills.

For our case study, we have selected archaeological sources of evidence that capture these two aspects of civic organisation. Based on this, we investigate the spread of Romanisation as an aspect of the evolution of the Roman infrastructure. We develop a mathematical model that utilises (sparsely) preserved sources of temporal information on the settlement system to compute a probabilistic activation sequence for the individual roads of the Roman overland transport network. A number of studies have been focusing on understanding the importance of Roman transportation networks for cultural, economical and political organization of societies [28, 32–34].

Our work builds on this research by considering the temporal evolution of the transportation networks. More specifically, given historical dates on municipalities that went through a formal civic act of Romanisation (more details on this follow shortly) during a particular time period, we obtain a probability for the activation of the roads connecting these municipalities to others in the settlement network. Note, however, that our model does not aim to reconstruct historical events or processes. In particular, we do not explore the spatio-temporal expansion of past societies, nor do we provide explanations for the connectivity structures in geo-archaeological landscapes, including how settlements were formed or why particular roads were built between them. Rather, the model has been designed to explore scenarios of road activation to help establish a fixed temporal framework in a complex spatio-temporal dataset that contains many significant archaeological observations at well-defined spatial locations but few reliable points in time. Our model is rather generic and allows for modifications to make it transferable to other archaeological scenarios.

The paper is organised as follows: First, in Section 2, we present a comprehensive overview of Romanisation as a historical process and its facets relevant for the socio-cultural change in ancient Tunisia. Specifically, we focus on the Roman settlement system and its interconnecting road network, which is supported by an extensive collection of archaeological evidence. Next, in Section 3 we introduce our mathematical model. Then, in Section 4 we apply our model to the case study of Romanisation in ancient Tunisia, calibrating it to the available archaeological data. To provide a proof of concept for our approach, we validate our model and perform sensitivity and stability analysis. Finally, we discuss our findings and possible future directions in Section 5.

## 2 Background: Romanisation as historical process

From a formalistic point of view, the Romanisation of the ancient Mediterranean world is an example of cultural diffusion ([35–38], etc.). From a historical point of view, however, it is an extremely important phenomenon, with effects that shaped successive civilisations until the present day (see e.g. [28, 39, 40] on the lasting economic effects of Roman trade infrastructure; [41] on military tradition; [42] on legal heritage). What is more, a political entity with pre-industrial means of communication and territorial control must have required a degree of loyalty throughout its provinces that could only be effected through profound social integration and a comparatively high standard of living [43, 44].

In this context, northern Africa is of particular (and long-standing: [45, 46]) interest, since it is a region that had a rich history of civilisation prior to the Roman conquest, yet prospered as a kind of fusion culture under the Roman rulers [47]. In fact, the region experienced unprecedented prosperity and population growth (mainly based on its proximity to the Roman heartland, its agricultural potential: [48–51]; but also supported by significant olive oil, pottery and textile industries, as well as marble-rich quarries: [52–55]), resulting in an immense density of Roman ruins within the territory of present-day Tunisia.

We will, to reinforce the notion, not attempt to tackle all historically relevant aspects of Romanisation in our area of interest. Instead, the data we selected serves as (partial) proxies for the dynamics of a much more complex whole. In this way, we reduce an intractably complex phenomenon of cultural diffusion to something more manageable by a compact and intuitive mathematical model.

### 2.1 Motivation and rationale

Our primary motivation is to provide a tool for extracting a plausible spatio-temporal backbone from our fully space-ordered and sparsely time-ordered data. Further aspects and connections of the immensely rich archaeological record could be explored in a more meaningful way, once such a primary structure was established. We decided to use mathematical modelling of time dynamics in a way that would be comprehensible and appropriate with regard to the temporal uncertainty in typical archaeological data. Our rationale in seeking a suitable solution was that even the most complex cultural diffusion process must be structured in space and time by some kind of constraints.

However, in order to extract any meaningful historical observations from the manifold archaeological manifestations, there must be some working knowledge of the underlying spatio-temporal dynamics. Unfortunately, while the spatial pattern of Roman settlement activity is easy to establish, thanks to numerous discovered sites and monuments, the temporal dimension is much harder to disentangle. There is a relative scarcity of precise time information (archaeological dates), which makes it difficult to understand the sequence and structure of historical change. On the other hand, the archaeological record should still provide enough information to produce plausible models of space-time dynamics and test their validity. Following these arguments, here we focus only on the Roman land transport network and we do not consider maritime mobility. For more details on the effect of sea travel on cultural and social interaction see [56, 57].

The speed and direction of cultural diffusion in antiquity can be assumed to have been constrained primarily by the challenges that topography and terrain posed to the movement of people across the landscape. The remains of Roman roads (many still physically present in the landscapes of Europe, the Near East and northern Africa; see [30] for a recent collection of case studies) have long been of particular interest to scholars. Their presence in any region’s archaeological record indicates a hitherto unknown capacity for managing resources and dividing labour effectively, marking the transgression to a higher level of state organisation and economic prosperity (see [58]).

Consequently, the Roman road network defines the primary spatial structure of the region’s settlement system and constitutes the first essential source of information for our study. The second one is provided by the Roman civic registers, which contain calendar dates for administrative status changes of several municipalities, thereby introducing a time axis into the analysis. True to the nature of archaeology, all of this evidence is partial and laden with uncertainty. The approach taken here is to use the data available transparently and to the maximum extent, so as to lend plausibility to our model’s design and outputs. We will give appropriate consideration to aspects of model validation when discussing the results of our work.

### 2.2 Archaeological setting

**Fig 1:**
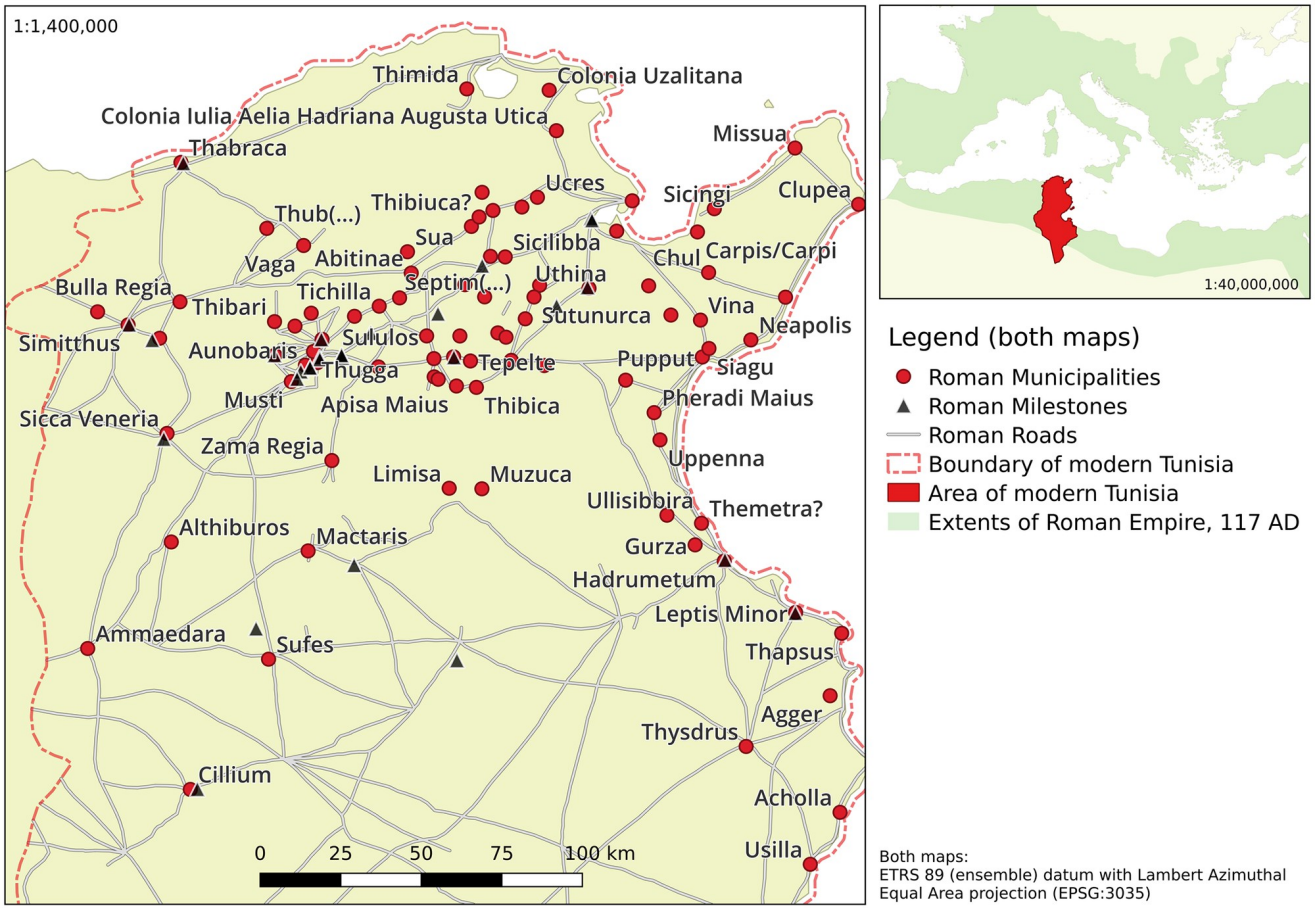
Location of area of interest (smaller map) and geographical distributions of selected municipalities and milestones used in this study, along with Roman road network. See sections 2.3 and S1 Appendix 4 for details. Third party data sources: Background topography by Natural Earth Project [59] (public domain); Roman roads network lines reproduced with permission from Ancient World Mapping Center [60] under a CC BY licence, original copyright 2015.

After the annexation and destruction of Carthage by Rome in the final year of the Third Punic War (149–146 BC), control over northern Africa was passed from Carthage to Rome. In the centuries that followed, a thriving Roman Republic and later Empire continued to push into the continent’s interior, reaching its greatest southern extent around 117 AD (see Fig 1), and eventually consolidating its territorial acquisitions in the province of Africa Proconsularis. The central, most populous and prosperous, part of the latter coincides geographically with modern day Tunisia.

The Romanisation of ancient Tunisia was a complex phenomenon that involved many aspects and strata of society. Here, we focus on the Roman era’s settlement system, composed of farmsteads, villages, towns and cities (collectively referred to as municipalities), and the roads connecting them (see [61, 62] for a more comprehensive discussion of the Roman settlement system in North Africa; see [24] for a detailed look at the region of modern-day Tunisia; also [58, 228–230], [63].

The density and splendour of Tunisia’s visible Roman ruins motivated the creation of fairly complete archaeological site catalogues by the end of the 19th century [64]. Following independence from France, heavy construction activities across the country necessitated rescue excavations in the 20th century that have resulted in more detailed studies of numerous sites [13]. As a result, there is ample information on site locations as well as their associated attributes of size, internal structure and function within the Roman era settlement system.

The Romans both founded new urban centres and developed existing ones: [65]. Along with this came a reconfiguration of the agrarian hinterlands, to effect the highest possible yields: [52, 66]. Indeed, the Roman alterations to some aspects of the settlement system of North Africa were so profound that some authors talk about ‘urbanisation’ as such when referring to them (e.g. [67]).

Transport infrastructure, in this case the overland roads network, determines the spatial structure of social diffusion (e.g. [68–70]). Throughout the former territories of the Roman Empire, several sources of evidence on the ancient road networks have survived (see local and regional studies, e.g.: [25, 72–74]; for details on the measure and construction of Roman roads: [40]):

- Road segments discovered within surveyed and excavated areas of archaeological sites,
- road segments (partially) observed in open country,
- ancient roads known to have survived underneath modern ones that still follow their courses (e.g. [25, 74]),
- roads known from ancient itineraries, travel reports and other literary sources
- observations of milestones, way stations, bridges and other remains that indicate the (former) presence of a road.

All of the above listed sources of evidence can be found in our area of interest ([75–77] 24 roads are mentioned in the Antonine Itinerary, and another six in Peutinger’s Table) but the preserved milestones constitute the most extensive source of information [13, 179].

To provide an idea of the original scale of the Roman road network: [78] suggests a total road length of 100,000 km, complimented by 8000 milestones and more than 1000 bridges (not that the system of coastal transport (ports) was no less well-developed [79], however, we consider this aspect out-of-scope for our present study).

In addition, the archaeology of Africa Proconsularis is exceptionally rich in Roman era inscriptions [13, 80]. Most pertinent to our case study, there is direct evidence on the hierarchical development of the settlement system in the form of status decrees (see also [15, 21–66]), [61, 297–301], [62, 44–49]. The municipalities of the Empire’s provinces could acquire different grades of self-government and jurisdiction by administrative decree. This was true for new settlements, established in conquered territories, as well as for existing ones. The settlement hierarchy of the study area can be summarised, very schematically, as a tripartite taxonomy of status (from highest to lowest ranking):

Colonia (pl. coloniae) was initially a strategically located outpost, established by the Romans in their conquered territories and inhabited by Roman citizens and veterans (a practice that was most pronounced during the first and ceased during the second century AD). Later, the name came to be used for those municipalities that stood on top of the settlement hierarchy in the Roman provinces. In terms of government, a colonia was an independent municipality with tax exemption from Rome, although its chartered (administrative) organisation eventually underwent changes. In general, the inhabitants of a colonia had full Roman citizenship.
Municipium (pl. municipia) was initially a municipality whose inhabitants were subject to all duties to Rome (e.g. military service, taxation), but did not enjoy all of the Empire’s civic rights (such as voting). All municipia had a certain amount of autonomy and were granted some rights to self-government, while remaining subordinate to Rome.
Civitas (pl. civitates) was a semi-autonomous municipality. Civitates featured different forms of administrative structure (e.g. civitas liber, civitas stipendiaria). Roman civil rights were not necessarily granted to their inhabitants, but could be awarded individually.

Civitates and municipia could be promoted to higher status (in our study area, no evidence for a demotion in status was found), and the preserved inscriptions of the related administrative decrees provide the most important source of temporal information for our study. In time, this hierarchy of status, and its implications for the populations of the provinces, became less well-defined. Eventually, the Constitutio Antoniniana (212 AD) granted all free men and women of the Roman Empire their associated Roman rights, which rendered the system of municipality status mostly redundant, as far as everyday life was concerned.

Regarding the Roman road network, contemporary evidence for the construction or maintenance of its individual segments can be found in the inscriptions of milestones. A milestone (miliarium, pl. miliaria) is a stone column or slab that was originally placed alongside a Roman road. Inscriptions on milestones recorded the distance from the starting point of the road (caput viae), counted in Roman miles (mille passus; a Roman mile is equivalent to 1000 paces or 5000 Roman feet, which equals about 1.481 metres).

In addition, milestones recorded the name of the official in charge of road construction or repair, and the name of the contemporary Roman ruler (by the 3rd century AD, milestones mostly served the purpose of commemoration: [81, 82]). Thus, milestones represent another, direct source of temporal data. In addition, if found in locations where no observable remains of the Roman road network have been preserved, milestones serve as proxy evidence for the presence of a road segment. However, due to the effects of weathering (erosion) on the stones’ surfaces, inscriptions might be only partially preserved. As a further limitation, not all milestones were found *in situ*, i.e. in the same place where they had originally been erected. Although the Roman occupation of modern day Tunisia began in 146 BC, the earliest Roman road construction, as evidenced by milestone inscriptions (e.g. CIL VIII, 10018) can only be dated to 14 AD.

Further aspects of Romanisation can be found in the area, which are secondary to the modelling approach presented here, but do represent sources of auxiliary temporal information. E.g., the Punic deities were aggressively supplanted by the new rulers, replacing them with or absorbing them into (*interpretatio romana*) the Roman pantheon. This process of religious reshaping is visible in newly erected, Roman-style temples, and its time dynamics are captured by consecration inscriptions. Intriguingly, the area is also home to an early Christian community, represented by a dense network of bishoprics, whose locations and founding dates are known through the records of a series of synods (Council of Carthage). However, this early entrenchment of Christianity represents a cultural change that was not an official trait of the Roman Empire until 380 AD, when Theodosius I began to forge Christianity into a unified state religion.

### 2.3 Data compiled for the study

The archaeological remains of the Roman era settlement system are most densely distributed in Northern Tunisia (roughly the area between 8°E and 11.5°E, and between 34.9°N and 37.5°N: see Fig 1), corresponding to the modern day population distribution of the country. It is a persistent pattern that can be explained by landscape properties: Whilst northern Tunisia is characterised by a mild, Mediterranean climate and fertile soils (particularly along the coast), the southeast is covered by salt-lake savannas (about 23% of the modern nation’s territory) and the Sahara desert (about 40–45%).

Data has been compiled from descriptions and observations of architectural remains and ancient texts from the Roman era, as published in printed works and online databases. Latin inscriptions and documents (e.g. letters, contemporary reports, decrees and other administrative texts) were a source of special significance for establishing the temporal data dimension. Precise datings (to a year or decade) are rare, but inscriptions mentioning people and events, that can be found on media such as milestones, provide sufficiently accurate date ranges for the purposes of this study. Further details on how this data was collected and curated, as well as references to all original sources are presented in the S1 Appendix.

For this study, we used a selected subset of Roman era settlements that are:

- situated within the area of interest between 8°E and 11.5°E, and between 34.9°N and 37.5°N,
- situated alongside a Roman road,
- associated with direct evidence that they had obtained a Roman civic status between 146BC and 400AD.

In total, 88 settlements (municipalities) were selected for analysis (see the instructions contained in [83] for identification of the exact subset used here). The time period of interest (146BC to 400AD) has been divided into nine discrete time frames. The first one lasts from 146BC until 0AD. The remaining eight represent the consecutive 50 year periods between 0AD and 400AD. We denote these time frames by *t* = 0 for the period from 146BC until 0AD, *t* = 1 for the period from 0AD until 50AD etc. For each settlement, we have nine data points that indicate whether the settlement is known to have had a Roman (civic) status in the respective time frame, and if so, which one. If the status is known to have changed more than once in a particular time frame, then we consider only the highest obtained status. We call the first time frame in which a Roman status is known the ‘time of Romanisation’ of a settlement (for simplicity’s sake, and disregarding the possibility that other manifestations of Romanisation might have taken hold here earlier).

The tasks of synthesising the evidence, and reconstructing the backbone of the road network, has already been accomplished for our area of interest as part of the monumental Barrington Atlas ([84]). This study uses the freely available, digitised derivatives in unmodified form (source: [85]).

Additional data, that was used in our study, consists of 29 milestones (plotted in Fig 1). The selection of milestones was based upon the availability of a date and the location alongside the investigated Roman road network. Milestones within the area of interest, but without clear affiliation to the given road network were dismissed for the analysis. Since precise dates for the erection of milestones are not available, we set two dates between 0 and 400AD for each milestone that define the time window of its potential erection.

To allow full reproduction of our methods and results, and to support further research, the data is freely available online (see [83]).

## 3 Method: Romanisation as road activation

The cultural phenomenon of Romanisation, in its multi-faceted nature, defies holistic attempts at modelling or simulating its spatio-temporal characteristics. Military, commercial, religious, and multiple other drivers (on the side of the native populations as well as the Roman conquerors) might have accelerated, slowed or sometimes even counteracted the diffusion of cultural traits.

Consequently, the traditional scholarly perspective on the Romanisation of North Africa is one of evolving political interests and directions. After the end of the Roman Republic, the Roman Empire began to focus on civic and commercial development on a grand scale, including the founding of numerous new municipalities. These continued investments would eventually make North Africa an exceptionally prosperous Roman territory, with a well developed road network as its defining infrastructural feature.

Since a complete representation of the Romanisation process in northern Africa is intractable, we turn to a reduced representation that still captures its essential spatio-temporal components. Having identified robust proxies in the remains of the Roman settlement system, its municipalities, its roads, and attributes associated with them (e.g. status decrees), we will next introduce a mathematical model of Roman road activation process. Our model is based on the principles of epidemiological diffusion models, and is given in a general form, wherefore we deem it transferable to other archaeological scenarios.

### 3.1 Model design and basic concepts

Our model incorporates the following design decisions, aimed at maximising information use and providing meaningful results:

**Simple input:** The model should not require any data or parameters that would be exceedingly difficult or costly to provide for typical archaeological use cases.
**Transparency:** Neither the computations nor the structure or mathematical concepts that constitute the model should be so complex as to make its working principles opaque and its output hard to interpret.
**Intuitive output:** Model output should be straight-forward to visualise and understand.
**Transferability:** It should be possible to apply the model, in essentially unaltered form, to a large variety of archaeological case studies.

A key idea in our model’s design is to transfer the temporal information from the settlement system to the temporal evolution of the Roman road network. More precisely, we use temporal data on Romanisation of municipalities to obtain the so-called ‘road activation’ probabilities. Since the status of municipalities is often changing in time, the road activation probabilities can also change in time. In this way, we can study the evolution of the road network under the influence of Romanisation. Mapped back into geographical space, the road activation process indicates a hypothetical spatial diffusion of the associated Romanisation process.

It is important to note that the activation time of an individual road does not necessarily represent its original construction date. Many roads and road segments can be assumed to have existed before the Romans took over maintenance, improvement and extension of the region’s infrastructure. Therefore, ‘activation’ refers to the integration of a road into the Roman era’s network; be it by first construction, repaving or continued use in unaltered shape. Our assumption on how the Romanisation process and road activation dynamics were coupled indicates that our model doesn’t consider the temporal evolution of all Roman roads, but only of those that were directly coupled to the temporal Romanisation data at hand. Also, we don’t consider roads which were built between already Romanised municipalities, e.g. for the purpose of infrastructure, but focus only on the newly Romanised municipalities. Additionally, we assume that at time 0 all road segments are assumed to be inactive, i.e. roads which already existed before the first time frame are not considered. Finally, any road-segment can be activated only once, i.e. road segments that became active can not be inactive later. Nevertheless, the probability of their activation can increase in time due to new Romanisation events.

Our model’s basic mechanics represent a Susceptible-Infected (SI) epidemic spreading process [86] in which non-Romanised municipalities are denoted as susceptible, and Romanised ones as infected. Note that we do not distinguish between municipality ranks (see Introduction). We only consider whether a town or a city gained an official Roman civic status or not. Also, since we are mainly interested in how the Romanisation spreading process triggered the road activation process, we only consider the ‘infection’ dynamics that lead to a municipality becoming Romanised (in the sense of an SI model) and not the ‘recovery’ process, when they were ‘de-Romanised’ (in a historical sense; after the Roman Empire’s withdrawal from northern Africa).

We build on the Cascade Transmission Model [87] and Independent Cascade Model [88], to model Romanisation as a stochastic process (**X**(*t*))_*t*∈**N**_ over a series of consecutive steps (time frames) that represent the temporal component of the diffusion pattern. In the initial time frame *t* = 0, we denote by $C_{0}$ the set of municipalities that were Romanised (infected) by then, i.e. until 0 AD. In all succeeding time frames, the likelihood of infection (transmission of Romanisation) of the remaining susceptible municipalities will depend on the strength of influence (that can be determined by, e.g., physical distance and rank) of previously Romanised municipalities.

In particular, we assume that in each time frame a Romanised municipality *p* influences, any non-Romanised one *c* independently, with a probability proportional to an influence *π*(*p*, *c*).

In Section 3.4, we will discuss different data-driven choices for the influence function *π* and how they affect the spreading process.

In each realisation of (**X**(*t*))_*t*∈**N**_, every non-Romanised (susceptible) municipality *c* can only be influenced by exactly one Romanised (infected) municipality *p*. On the other hand, an infected municipality *p* can infect (given that its strength of influence is sufficient for a successful transmission) more than one susceptible municipality per time frame. In the following, we will introduce how each realisation of the modelled process generates a time-evolving, directed spreading tree. For this, we will use the terminology from graph theory:

- initial nodes $C_{0}$ are called *root nodes*;
- if *c* gets Romanised under the influence of *p*, then *p* is called *a parent* of *c* and *c* is called *a child* of *p*;
- when *c* gets Romanised under the influence of *p*, *a directed edge* (*pc*) is added to a spreading tree.

**Fig 2:**
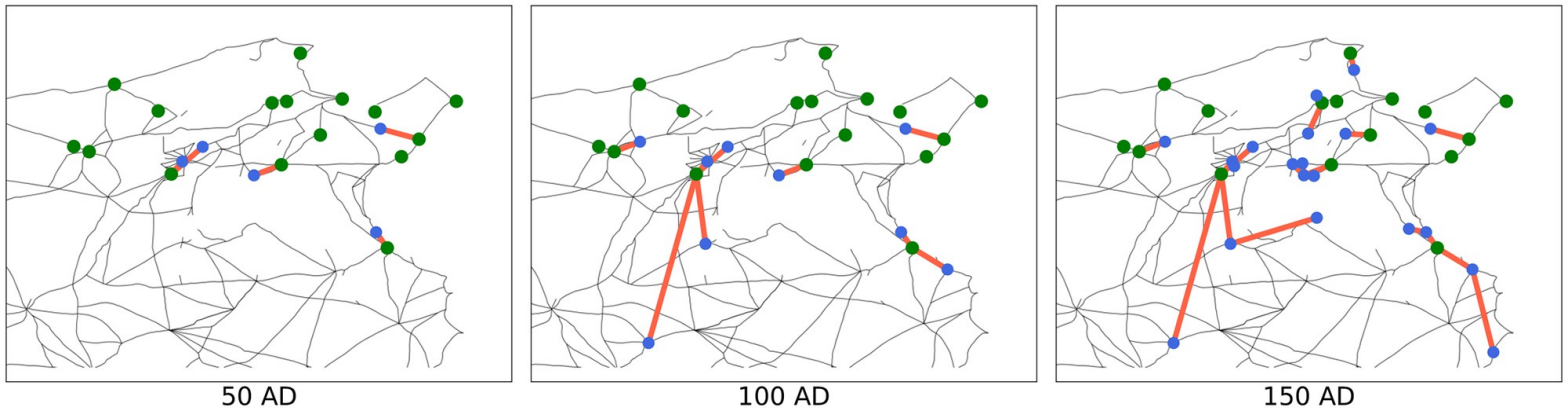
Temporal evolution of one realisation of the spreading process represented as a spreading tree. Green nodes indicate the initially Romanised municipalities at 0 AD and blue nodes municipalities that have a Roman status in the respective time frame. Each connected component of the spreading tree is a tree rooted in a green node. These trees indicate how the municipalities got influenced. Roman roads network lines reproduced with permission from Ancient World Mapping Center [60] under a CC BY licence, original copyright 2015.

The nodes of the spreading tree in any given time frame are the municipalities which are Romanised in that time frame and directed edges indicate how each of these was influenced. In Fig 2 we show one possible realisation of (**X**(*t*))_*t*∈**N**_ as a time-evolving spreading tree. Once all municipalities have been Romanised, every one of them is part of the spreading tree. Hence, it becomes a directed spanning tree (more precisely a forest, since we have several roots) that contains the information on how the spreading of Romanisation took place in that particular realisation. We call this directed spanning tree *the cascade* [87], while in epidemiological context, this is often called the infection chain [89].

Next, we will show how above introduced concepts can be used to obtain probabilities that a municipality *c* gets Romanised under the influence of *p*, constrained under the given data. Let $S_{0}$ be the set of cascades on the nodes C whose set of roots is given by $C_{0}$. Since any realisation of the process generates an individual cascade in $S_{0}$, we can define a random variable *S* taking values in $S_{0}$ that resembles the cascade of the process.

However, not all realisations of the process are historically feasible, i.e. consistent with our data. The data, which we abstractly denote by D, provides us with the time of Romanisation *t*_*rom*_(*c*) of every municipality c∈C. The time of Romanisation $t_{rom}\left(c\right)\in \left\{0,\ldots ,8\right\}$ is the first time frame for which a Roman municipality status is known. In particular, D defines the order in which the municipalities got Romanised and thereby greatly restricts what values *S* can take, given D. We call $s\in S_{0}$ historically feasible if for any edge (*pc*) ∈ *s* we find *p* ∈ *P*_*c*_, where

$$\begin{array}{c} P_{c}=\left\{p\in C\;|\;t_{rom}\left(p\right)<t_{rom}\left(c\right)\right\} \end{array}$$

is the set of possible parents of c∈C, s.t. *P*_*c*_ = ∅ for $c\in C_{0}$. We denote the set of historically feasible cascades by $S_{0}^{h}$ and note that by definition

$$\begin{array}{c} P(S\in S_{0}^{h}\;|\;D)=1. \end{array}$$ (1)

We are interested in a probabilistic analysis of *S*, given D. In particular, for c∈C and *p* ∈ *P*_*c*_ we want to compute the probabilities

$$\begin{array}{c} \theta_{p,c}≔P((pc)\in S\;|\;D), \end{array}$$ (2)

where *θ*_*p*,*c*_ is the probability that *c* gets Romanised by the influence of *p*, based on the available data D, i.e. the information that *c* got Romanised at time *t*_*rom*_(*c*). As discussed above, our model assumption is that the probability that a municipality *p* influences *c* is proportional to *π*(*p*, *c*), thus we set

$$\begin{array}{c} \theta_{p,c}=\frac{\pi (p,c)}{\sum_{p^{\prime }\in P_{c}}\pi \left(p^{\prime },c\right)}. \end{array}$$ (3)

By the independence of the spreading events, the *θ*_*p*,*c*_ are independent from one another for different *c*. Thus, we can compute the probability that the spread of Romanisation occurred along a particular cascade $s\in S_{0}^{h}$ by

$$\begin{array}{c} P\left(S=s\;|\;D\right)=\prod_{(pc)\in s}\theta_{p,c}. \end{array}$$ (4)

It can be shown that $\sum_{s\in S_{0}^{h}}P\left(S=s\;|\;D\right)=1$, but due to the extremely high number of all possible cascades, the probabilities of individual cascades are very small. However, in this paper the probabilities of specific cascades are not of major interest, but rather the edge probabilities *θ*_*p*,*c*_ which indicate how likely it is that Roman influence spread between two municipalities.

### 3.2 Representation of Roman road network

In the previous section, we introduced concepts for studying the Romanisation process between municipalities. However, our main goal is not to recover the spreading process itself, but to determine the activation probabilities of Roman roads governed by the spreading process. Thus, we will construct an appropriate representation of the Roman road network and then, in Section 3.3, we will project the information on the cascades between municipalities to this network, in order to determine the Roman road activation probabilities.

We obtained a cut-out of the Roman road network of ancient Tunisia from [85] and rasterised it to obtain a simple data structure with just enough spatial accuracy for the purposes of our model. Based on this, we will construct a network G=(R,E), with a set of nodes R and a set of edges E.

Since network extraction from a raster (image) file is not the main research focus of this paper, we introduce here a basic extraction approach. For possible further improvements of our approach, we refer interested readers to the following recent publications [90, 91].

**Fig 3:**
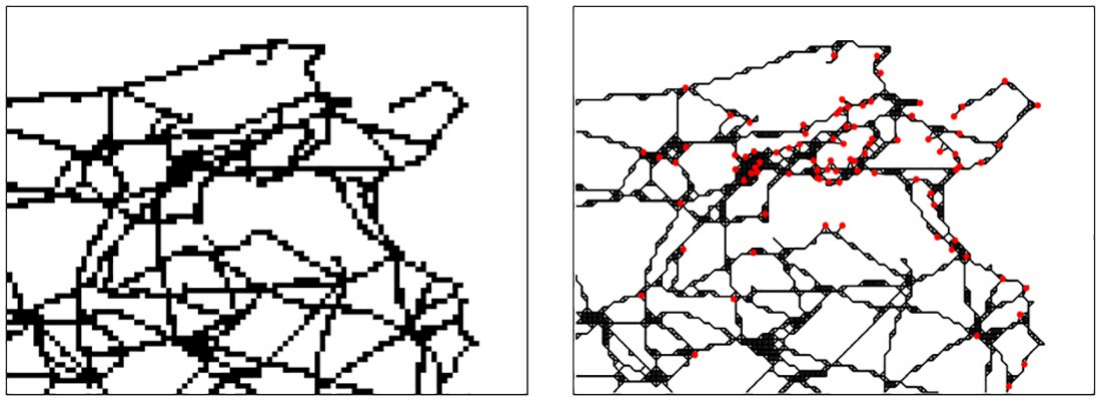
Roman roads in ancient Tunisia. Left: Scaled down image of the Roman road network. Right: The Roman road network, where red nodes resemble settlements and black edges road segments. Generic road nodes are omitted. Sources: Natural Earth [59]; Ancient World Mapping Center (AWMC) [60, 85]. Roman roads network lines reproduced with permission from Ancient World Mapping Center [60] under a CC BY licence, original copyright 2015.

In order to reduce complexity, we scale down the road map raster from its original resolution of 1204 × 897 to a 90 × 73 pixel image, which can be seen in the left picture of Fig 3. We transform this image into a network which consists of a node for each black pixel and an undirected edge between any two neighbouring pixels (we consider 8 neighbours). We call these nodes *generic road nodes*. To embed the municipalities into this network, we include an additional municipality node (m-node) for each municipality and connect it to the four closest generic road nodes (r-nodes) that lie inside a radius of 8 km. If there are no r-nodes within this radius, the m-node will not be added to the network and is not considered in the model. The resulting Roman road network is shown in the right picture of Fig 3. Since each r-node has a geographical location that can be derived from its position in the image, and the geographical locations of the municipalities are also given, we can assign a geographical distance to each edge.

This procedure constructs a weighted network *G*, that we will call Roman road network, and it consists of the following components:

*m-nodes*: the 88 settlements (municipalities) considered in our analysis, c∈C.
*r-nodes*: 1426 nodes that represent the course of the Roman road network. There is an r-node for each pixel in the left image of Fig 3.
*road segments*: the edges between network nodes (m-nodes and r-nodes), e∈E. A road segment either connects two r-nodes or connects an m-node to its neighbouring r-node.

In total, our constructed Roman road network *G* has 1514 nodes (|C|=88 are m-nodes and 1426 are r-nodes) and |E|=3132 edges, i.e. road segments. The road segments will be the central objects of interest in the next Section 3.3, when inferring the temporal activation sequence of the road network *G*.

### 3.3 Temporal activation of road segments

So far, we used civic status changes and their associated temporal information as key indicators of Romanisation spreading from one municipality to another. More precisely, in Section 3.1 we derived probabilistic statements on temporal connections between municipalities, e.g. *θ*_*p*,*c*_. Here, we will use these statements to infer to which extent the Romanisation process affected the temporal activation of the Roman road network *G*.

**Fig 4:**
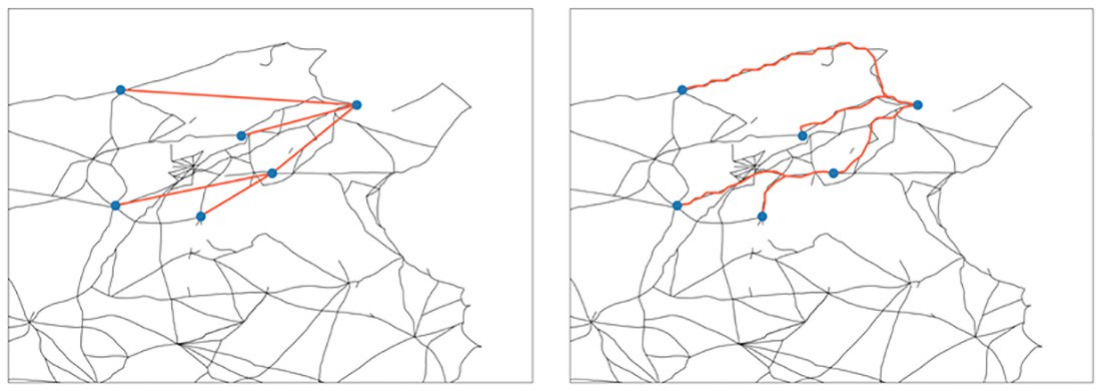
Left: Example of a spreading tree. Right: Roads in the Roman road network that correspond to the spreading tree shown in the left figure. Roman roads network lines reproduced with permission from Ancient World Mapping Center [60] under a CC BY licence, original copyright 2015.

The road activation process will be defined such that in each time frame road segments, i.e. edges of *G*, can be active or inactive. We assume that road activation was governed by the Romanisation process, i.e. road segments were activated to connect newly Romanised municipalities to the network. In order to formally connect the road activation dynamics and the Romanisation process (**X**(*t*))_*t*∈**N**_ (as introduced in Section 3.1), we will project the temporal connections between municipalities *θ*_*p*,*c*_ to connections on the road network *G*. In Fig 4 we show one example of connections between municipalities, using a spreading tree and their corresponding projection to the road network *G*.

As derived in Section 3.1, a connection (*p*, *c*) between a municipality *c* that was influenced by a previously Romanised municipality *p* ∈ *P*_*c*_ was formed with the probability *θ*_*p*,*c*_. When projecting this to the road network, to each pair of municipalities c∈C and *p* ∈ *P*_*c*_, we now associate a path Γ_*pc*_ from *p* to *c* in the Roman road network *G*. A path Γ_*pc*_ consists only of two m-nodes *p*, *c* and r-nodes. In our model we choose Γ_*pc*_ to be the shortest path from *p* to *c* in *G*, computed using the Dijkstra’s algorithm [92]. Due to the grid-like structure of our network, the shortest path is not always unique. However, different shortest paths between municipalities only differ in few places by a small amount of road segments, such that the course of the shortest path is overall unique. The results of our model have proven to be robust against different choices of shortest paths.

**Fig 5:**
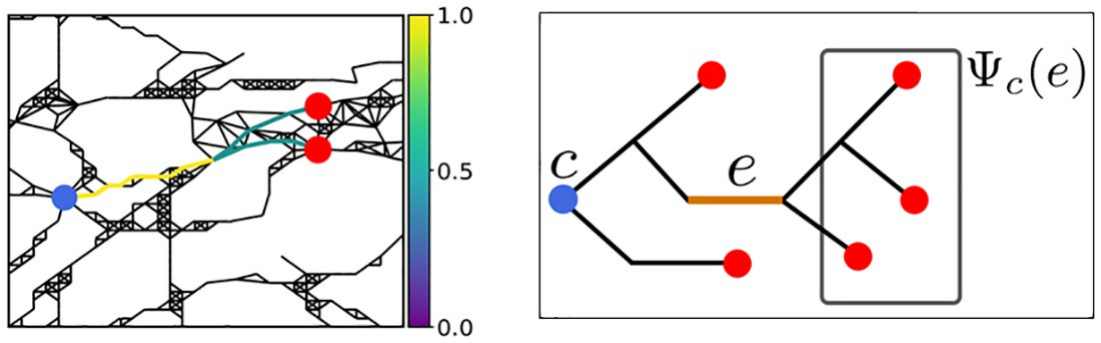
Projection to the road network. Left: Probabilities of example road segments indicated by the colour map. Municipalities on the right were Romanised before those on the left. Right: Schematic illustration of the road activation procedure. Roman roads network lines reproduced with permission from Ancient World Mapping Center [60] under a CC BY licence, original copyright 2015.

Romanisation of *c* by *p* in a time frame *t* initiates the activation of all road segments in Γ_*pc*_ in that time frame. Hence, a road segment *e* gets activated in the Romanisation event of *c*, i.e. at time *t*_*rom*_(*c*), if *e* lies in the shortest path from *c* to its parent. The probability of this event is

$$\begin{array}{c} \phi_{c}\left(e\right)=\sum_{p\in \Psi_{c}\left(e\right)}\theta_{p,c}, \end{array}$$ (5)

where Ψ_*c*_(*e*) = {*p* ∈ *P*_*c*_ | *e* ∈ Γ_*p*,*c*_} is the set of all possible parents of *c* whose shortest path to *c* contains *e*, see the right plot in Fig 5 for a schematic illustration. Since $\sum_{p\in \Psi_{e}\left(c\right)}\theta_{p,c}\leq \sum_{p\in P_{c}}\theta_{p,c}=1$, it holds that *ϕ*_*c*_(*e*) ≤ 1 for all c∈C and e∈E. In particular, *ϕ*_*c*_(*e*) = 1 holds if all shortest paths from all possible parents *p* to *c* contain *e*. In the left plot of Fig 5 we show an example where there are two possible parents (marked in red) of one node (marked in blue). In our model, these situations are resolved such that the edges where both shortest paths coincide have activation probability one (marked in yellow). The other two edges have probability one half each (marked in blue), as from the available data it is not clear whether the lower or the upper road was activated.

Using these assumptions, we will next derive the expression for calculating the road segment activation probabilities at time *t*. Since any road segment can only be activated once, we will keep track of the probability *β*_*e*_(*t*) that *e* is *not* active at time *t*. Introducing the sets of municipalities which got Romanised before or at time *t*

$$\begin{array}{c} C_{t}=\left\{c\in C\;|\;t_{rom}\left(c\right)\leq t\right\}, \end{array}$$

we can compute the probability of a road segment *e* not being active at time *t* as

$$\begin{array}{c} \beta_{e}\left(t\right)≔P\left(e\;\text{is}\;\text{not}\;\text{active}\;\text{at}\;\text{time}\;\text{t}\right)=\prod_{c\in C_{t}}\left(1-\phi_{c}\left(e\right)\right). \end{array}$$ (6)

Here, we used the independence of the *θ*_*p*,*c*_ for different *c*, which implies that the probabilities *ϕ*_*c*_(*e*) are independent for different *c*. Finally, we obtain the probability of a road segment *e* to be active at time *t* by

$$\begin{array}{c} \alpha_{e}\left(t\right)≔P\left(e\;\text{is}\;\text{active}\;\text{at}\;\text{time}\;\text{t}\right)=1-\beta_{e}\left(t\right). \end{array}$$ (7)

Following the ideas presented above, we now present a step-by-step approach for calculating the activation probabilities of road segments. The pseudocode for our approach can be found in the S1 Appendix. The code for the model and all subsequent analyses is available at [93].

**Outline of our approach for calculating the activation probabilities of road segments**

1. Define influence function *π*(*c*, *c*′), see section 3.4 for more details.
2. For all municipalities *c* determine the parent set *P*_*c*_ of municipalities *p* Romanised before c.
3. Calculate shortest path Γ_*pc*_ with Dijkstra’s algorithm for all municipalities *c* and their parents *p* ∈ *P*_*c*_.
4. Calculate *π*(*p*, *c*) based on the choice from step 1.
5. Calculate *θ*_*p*,*c*_ according to (3).
6. Determine the probability *ϕ*_*c*_(*e*) of *e* getting activated by *c* according to (5).
7. Calculate *β*_*e*_(*t*) for all edges *e* according to (6).
8. Calculate the activation probability *α*_*e*_(*t*) for all road segments *e* according to (7).

### 3.4 Choice of influence function

The model presented above is quite general and can be applied in many ways. In particular, the influence function (as defined in (3)) can be adjusted for other data and scenarios. Here, we will introduce two possible choices of influence function and explain how they compare to each other.

- **Distance between municipalities:** It is natural to assume that municipalities that were close had a greater influence on one another than those that were far apart. To account for this factor, we define the influence *π*(*p*, *c*) for $c\in C,p\in P_{c}$ by some decreasing function *g*
  $$\begin{array}{c} \pi \left(p,c\right)=g\left(d_{p,c}\right), \end{array}$$ (8)
  based on the distance *d*_*p*,*c*_ between *p* and *c*. Even though it seems like the most natural choice, the geographical distance between municipalities might not be appropriate here. There are municipalities that are geographically close but are separated by rough terrain like mountains, so that travellers take a much longer route than the direct path. Therefore, we take *d*_*p*,*c*_ to be the length of the shortest path Γ_*pc*_ in the road network constructed in Section 3.3. This choice is justified under two assumptions. The first assumption is that the optimal, feasible path between two municipalities is part of the final Roman road network. The second assumption is that a path can be used by travellers even though the road has not been built yet in the model. This seems like a critical assumption. However, historically most paths already existed and were used before they were activated to become a Roman road. Hence, these paths could have been used by travellers and thereby exerting influence.
  The unit of *d*_*p*,*c*_ affects the values of the influence function. For most choices of *g*, scaling the distance will change the proportions of the influences and thereby alter the results of the model. Choosing $g\left(x\right)=\frac{1}{x^{\rho }}$ avoids this problem but requires a choice of a scaling parameter *ρ*.
  If introducing a parameter to the influence function is necessary, the problem of scale can be fixed for arbitrary functions *g*, by considering *π*(*p*, *c*) = *g*(*ρ* ⋅ *d*_*p*,*c*_) for *ρ* > 0. In our model we will use the influence function *g*(*x*) = exp(−*ρx*). The parameter *ρ* enables us to control how rapidly the influence decreases with increasing distance (also known as distance decay). Small values lead to higher influences (relative to the influences of municipalities that are closer) over greater distances, thereby rendering the distance less important. For values tending toward 0, all influences are the same, independent of the distance. Large values lead to a rapidly decreasing influence with an increase in the distance. For values tending to infinity, only the closest municipality will have an influence. Choosing this parameter is a non-trivial task and requires expert knowledge.
  In our network model, the distances are measured in kilometres and the average distance to the closest municipality is 13 km. We choose 1 to be a range in which the scaled distances lie, so we set the scaling parameter to be *ρ* = 0.08 and obtain the following influence function
  $$\begin{array}{c} \pi \left(p,c\right)=\text{exp}\left(-0.08\cdot d_{p,c}\right). \end{array}$$ (9)
  We observed that our results are robust against variations in this parameter.
- **Civic Status:** We can also include the temporal information about the Roman civic status in the influence function *π*(*p*, *c*). We assume that municipalities with a higher status had a greater influence. However, that status can change over time, while we describe the influence of *p* on *c* as a fixed value. This is not a problem, since in the model the influence of *p* on *c* only matters at the point in time when *c* becomes Romanised. Hence, we simply use the Roman civic status of *p* at time *t*_*rom*_(*c*).
  Quantifying the dependence between civic status and influence *π*(*p*, *c*) requires expert knowledge. As an example, we set the influence of civitates to be unchanged, apply a factor of 2 to municipia and a factor of 2.5 to coloniae. In this way, we assume that municipia have twice the influence of civitates. In order to be comparable with the standard model, we assign to municipalities with one or more unsure status their highest potential status. Let *σ*(*p*, *t*) be the status influence function of *p* at time *t*. If *p* is not Romanised at time *t* then we set *σ*(*p*, *t*) = 0. Thus, the status influence function we use in our model is defined in the following way

**Table:** 

**Status of** *p* **at** *t*_*rom*_(*c*)	civita	municipium	colonia
*σ*(*p*, *t*_*rom*_(*c*))	1	2	2.5
  We apply this factor to the previous influence function (9) to obtain
  $$\begin{array}{c} \pi \left(p,c\right)=\sigma \left(p,t_{rom}\left(c\right)\right)\text{exp}\left(-0.08\cdot d_{p,c}\right). \end{array}$$ (10)

Next, we will compare the results obtained by applying these two choices of influence function. Further extensions for the choice of *π*(*p*, *c*) could include information about the terrain, as well as different socio-political and cultural attributes of municipalities.

## 4 Results: Romanisation as analytical framework

In this section, we will analyse the outputs produced by our model and evaluate their accuracy with the help of archaeological findings of milestones that indicate the existence of a road during a particular time frame.

The output of the model is given in 8 discrete time frames, each corresponding to a 50 year period, i.e. *t* = 1 is 0–50 AD up to *t* = 8 which is 350–400 AD. Note that these time frame durations, and their starting point, do not correspond to meaningful historical events in our area of interest. They also do not contain the entire epoch of Roman presence and influence in the area. Rather, they represent a reasonable compromise between intuitive time units, sparse data and analytical accuracy, while capturing all of the temporal data available for the study. Following the approach in Section 3.2, the map of Roman roads is discretised into a network *G* with 1514 nodes and 3132 edges, the latter representing road segments. Then, for each of the road segments and each time frame, we calculate an activation probability as described in Section 3.3.

We consider only road segments that were part of the Roman road network, as given in the available archaeological records, and we do not infer other possible connections between settlements (m-nodes). Additionally, as discussed before, our assumption on the coupling between the Romanisation process and road activation dynamics meas that our model focuses only on the temporal evolution of Roman roads that are directly supported by our available temporal data. We capture the first municipality status changes around 0 AD, i.e. by comparing their status before 0 AD and in the period 0–50 AD. Such status changes can impact the activation of only those roads built in 0–50 AD, whose construction contributed to the Romanisation process. In particular, roads that were built between existing settlements, to enhance their infrastructure, are not considered in the model. Therefore, one needs to be careful with the interpretation of the results. A low activation probability is not necessarily to be interpreted as a high probability that the road did not exist.

Note that the strict limitation of our model to road network dynamics also implies limited explanatory power. In particular, pre-existing overland and maritime networks constitute hidden background variables in the analysis. Their latent influence is likely reflected in the data and might well have an effect on model outcomes. However, since these effects are not explicitly captured by our road activation model, we will exclude them from our interpretations (regardless of their historical plausibility).

### 4.1 Observations and discussion

We apply the method introduced in the previous section to compute the activation probability of the road segments by following steps 1–8 of our approach for calculating temporal activation probabilities (see 3.3).

To highlight the effects of different choices of influence function, we focus on the following two model variants:

- **Model 1:** where the influence function is dependent only on distances between m-nodes, i.e. *π*_1_(*p*, *c*) = exp(−0.08 ⋅ *d*_*p*,*c*_), where distances *d*_*p*,*c*_ refer to the shortest path distance in the road network measured in *km*.
- **Model 2:** where the influence function depends on distances but also on municipality status, i.e. *π*_2_(*p*, *c*) = *σ*(*p*, *t*_*rom*_(*c*))exp(−0.08 ⋅ *d*_*p*,*c*_).

**Fig 6:**
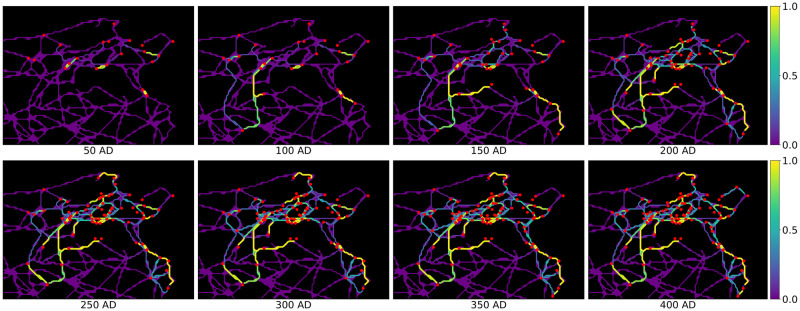
Temporal activation probabilities of the road segments. The influence function is chosen as in Model 1 by *π*(*p*, *c*) = exp(−0.08 ⋅ *d*_*p*,*c*_). Romanised municipalities are marked in red. Roman roads network lines reproduced with permission from Ancient World Mapping Center [60] under a CC BY licence, original copyright 2015.

**Fig 7:**
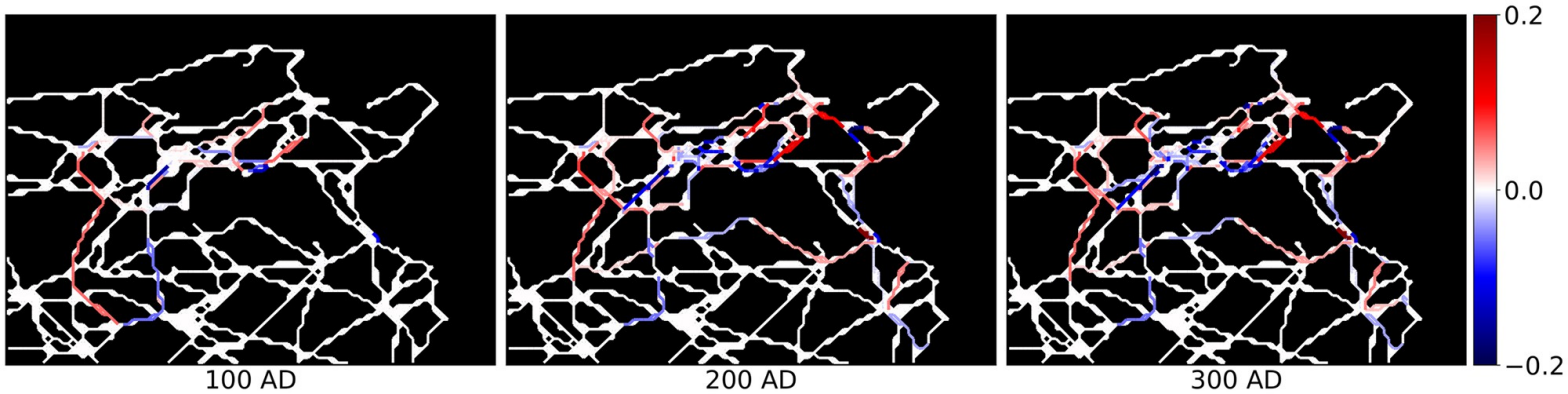
Shift in the road activation probabilities wrt. Model 1 when including the municipality status in the influence function. White roads denote a good fit between the two models, red roads indicate higher activation probability in Model 2 and blue roads higher activation probability in Model 1. Roman roads network lines reproduced with permission from Ancient World Mapping Center [60] under a CC BY licence, original copyright 2015.

For each of the two models, in step 8 we obtain temporal activation probabilities of all road segments over the 8 considered time frames. The resulting temporal activation probabilities of road segments for Model 1 can be seen in Fig 6, where the colour of each road segment indicates its activation probability in a given time frame. We can observe that main road patterns get established quickly (after 3–4 time frames) and that path dependency is clearly visible, i.e. there is no uniform spatial activation probability distribution of roads. The results also indicate more activation on the west coast than on the north coast, which is due to more administrative reorganisation in that area. This overall pattern also persists in Model 2, such that the results of the two models agree to a great extent. However, the impact of including the municipality status in the influence function in Model 2 is evident in our analysis. We demonstrate this effect in Fig 7, which illustrates the shift in the activation probabilities between Model 2 and Model 1. Here, white roads denote a good fit between the two models, red roads indicate higher activation probability in Model 2 and blue roads higher activation probability in Model 1.

We observe that in Model 2 roads surrounding densely connected regions have higher activation probabilities than in Model 1. These roads are marked in red in Fig 7. The reason for this is that many municipalities in these regions were coloniae, which in Model 2 have higher importance. Therefore, roads that connected them to other municipalities were activated with higher probability.

These observations lead directly to our interpretation of the model output in relation to the historical context of ancient Tunisia’s Romanisation. In the introduction of this study, we showed that there seems to be wide-spread consensus about road construction being a hallmark of Romanisation. The extent to which this coincided with a substantial reconfiguration of the settlement system itself depended on regional preconditions as much as on Roman strategic interest. In our area of study, dominant economic centres of the interior (such as Thugga), as well as ports of trade along the east coast (such as Hadrumetum), retain their pre-Roman significance and appear as hubs of connectivity in the sequence of road activation (Fig 6). These observations are in line with the results of the extensive settlement system analysis conducted by [61] for Roman North Africa. They provide further support for the view that the Roman rulers strengthened the existing settlement hierarchy, rather than making significant alterations to it.

Note that these observations appear to contradict the views of other authors, such as [67]. This can be explained by the multi-layered nature of settlement systems. Our observations only apply to the urban centres and the major roads connecting them. In contrast, the agrarian hinterland might have been subject to drastic reorganisation to optimise yield of the produce preferred by the Romans (see [49, 52]). However, the published archaeological body of evidence for that layer of the settlement system is too small to have a visible effect on our model results.

Overall, although the available temporal data is sparse, in conjunction with the denser spatial data and a model-based approach, it allows for the main patterns of connectivity to be identified. Furthermore, we showed how the model can be used to study different scenarios and in particular, how much the choice of the influence function can impact the modelled road activation process.

### 4.2 Model validation

**Fig 8:**
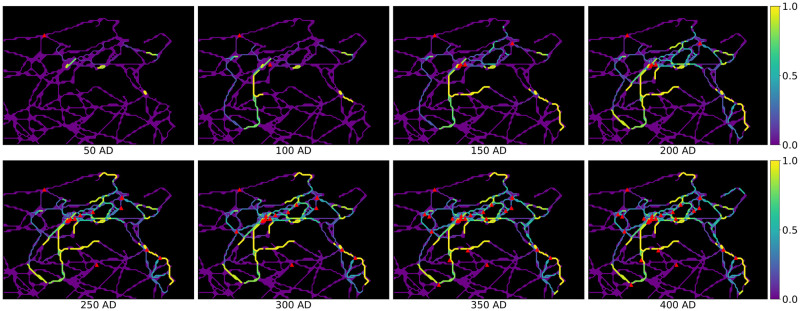
Temporal activation probabilities of the road segments obtained using the distance influence function, plotted together with milestones (red triangles). Roman roads network lines reproduced with permission from Ancient World Mapping Center [60] under a CC BY licence, original copyright 2015.

To validate our model, we are using the dataset of 29 milestones introduced in Section 2.3, that we consider to have in situ locations and accurate temporal information on when they were (most likely) erected. We assign every milestone to its associated road segment and consider the latter’s activation probability. A high activation probability of these road segments before or at the time the respective milestones were placed, indicates a good fit between our model and the validation data. We plot the activation probabilities together with the temporal milestones in Fig 8, i.e. in each time frame we plot only milestones that were placed before or during this time frame.

For each milestone, we assign all road segments within a radius of 4km to it. The radius of 4km is chosen wrt. to the discretised road map. In order to derive a single activation probability for a milestone in each time frame, we take the maximum over the activation probabilities of all road segments assigned to it. We take the maximum rather than the mean value, because there are many road segments that are not considered by the model and therefore have an activation probability of 0, and the arithmetic mean is not robust to extreme values.

**Fig 9:**
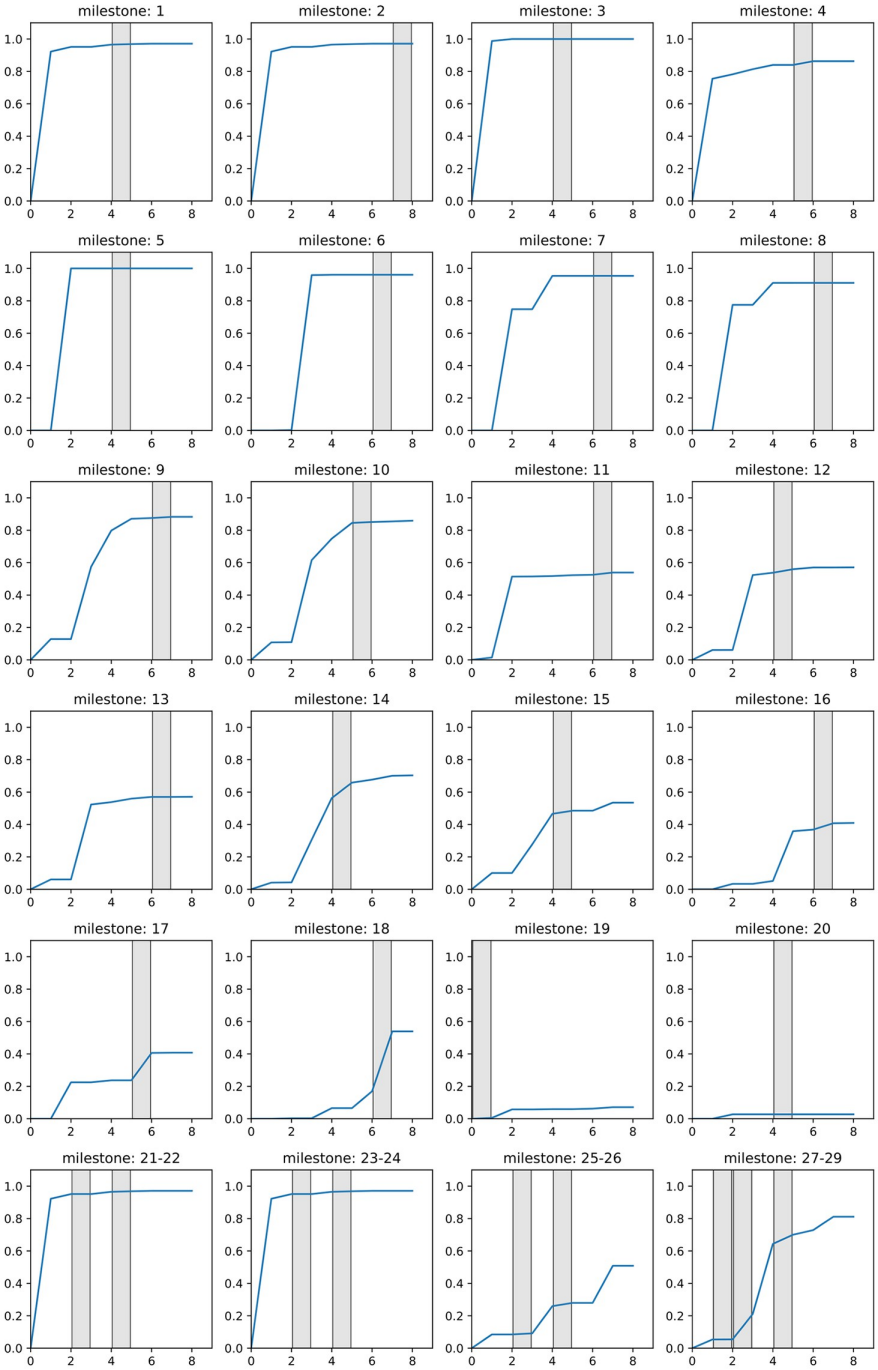
Temporal activation probability of the road segments around the milestones. Milestones that were found in the same location are grouped in one image. The time frames the respective milestones were found are marked in grey. Roman roads network lines reproduced with permission from Ancient World Mapping Center [60] under a CC BY licence, original copyright 2015.

We plot the temporal activation probabilities of the road segments around the milestones in Fig 9. The activation probabilities around milestones 1 to 18 each have their highest value before or in the time frame in which the respective milestone was erected. We sorted the milestones in the figure according to the shape of their activation probability graph. Namely, for milestones 1 to 10 the activation probabilities reach a value larger than 0.8 in the time frame in which the respective milestone was placed, showing an excellent fit between the validation data and our model output. Next, the roads around milestones 11 to 15 were activated with a probability of around 0.5. Although these activation probabilities do not reach very high values, these values are reached before or in the time frame the respective milestones were placed. However, according to our model, road segments around the milestones 19 and 20 get activated with a very small probability of less than 0.2. There are some explanations for this result: Milestone 19 was found around the city of Thabraca, which was a colonia in the first time frame. Since our model only infers roads which were part of the expansion of the Roman Empire after 0 AD, roads that would connect Thabraca to previously Romanised settlements are not considered. The small activation probability in later time frames comes from Thabraca being a parent to newly Romanised municipalities. Milestone 20 lies in the remote southern region, on roads that connect the western and eastern part of the area we are considering. Although such roads certainly played a big role in improving the infrastructure of the region, available data does not provide information about the Roman status of the municipalities there. Thus, these roads were not needed to connect newly Romanised municipalities to the road network and are activated with a probability close to 0 in our model.

Finally, in the last four subfigures we grouped milestones that were found at the same location, but were placed in different time frames. Since they were found at the same location, they are assigned to the same road segments and thereby share the same activation probability graph. Among other reasons, multiple milestones at the same location can indicate that the road next to them was fortified or repaired once or even multiple times. Here, we again see a good fit between our model and the milestone data for milestones 21–24. Most of the cites around milestones 25–26 were Romanised in the first time frame, so the results can be explained similarly to milestone 19. A very good fit can be observed for milestone 29, reaching an activation probability of 0.8. However, the activation probabilities around milestones 27 and 28, placed at the same location as 29, have a very small value at the time when these milestones were placed. The reason for this is that milestones 27 and 28 lie in the central northern region, where there are many densely spaced municipalities (see Fig 8, time frames correlating to 100 and 150 AD).

Since our model connects only newly Romanised municipalities to one of their closest parents, initially only two clusters (west and east) are formed, without activating the roads in the area between them, i.e. the area where milestones 27 and 28 are located. With a delay of 1–2 time frames, we observe the activation of roads connecting these two clusters.

While this analysis provides valuable insights into the model, it limits the quantification of a global model fit. A robust, integrated metric would enable comparison across models (assessing different mechanisms) and across case studies (evaluating different scenarios). However, defining an appropriate goodness-of-fit for such spatio-temporal models, applied to sparse data, presents a significant challenge due to the inherent complexities and will remain a topic for future research.

### 4.3 Sensitivity analysis

One of the main challenges of modelling historical processes is that available archaeological data is sparse and uncertain. Therefore, it is important to examine if methods for such data are robust to perturbations of the input data. The data set that we use in this paper is also prone to errors, especially regarding the time of Romanisation, that can in principle vary several years. In this section, we will analyse the impact that perturbations of available temporal data have on the results of our method. Large deviations in outcomes produced by small perturbations of the input data will indicate high sensitivity of our method, i.e. low robustness.

Given the nature of the historical process we study, we assume that the data uncertainty stems from a municipality being Romanised earlier than what the data shows and not later. Thus, for each municipality c∈C, we will perturb its time of Romanisation *t*_*rom*_(*c*) by

$$\begin{array}{c} {\tilde{t}}_{rom}\left(c\right)≔t_{rom}\left(c\right)+X,\;X∼\text{Bernoulli}\left(p\right), \end{array}$$

where we introduce the Bernoulli distribution with

$$\begin{array}{c} P(X=-1)=p,\;P(X=0)=1-p, \end{array}$$

and choose *p* = 0.2 in our experiments. In the case that ${\tilde{t}}_{rom}$ is less than 0 we set it to 0.

Next, we compute the activation probabilities of the road segment *e* at time frame *t* using the Bernoulli perturbed data $\tilde{D}$ by ${\tilde{\alpha }}_{e}\left(t\right)≔P\left(e\;\text{is}\;\text{active}\;\text{at}\;\text{time}\;t\;|\;\tilde{D}\right)$, based on Model 1. We compare ${\tilde{\alpha }}_{e}(t)$ to the *α*_*e*_(*t*) (with the original data D) using the mean squared error

$$\begin{array}{c} v_{e}\left(t\right)≔E[{\left(\alpha_{e}\left(t\right)-{\tilde{\alpha }}_{e}\left(t\right)\right)}^{2}], \end{array}$$ (11)

that describes the expected deviation from the results of the temporal activation probability.

**Fig 10:**
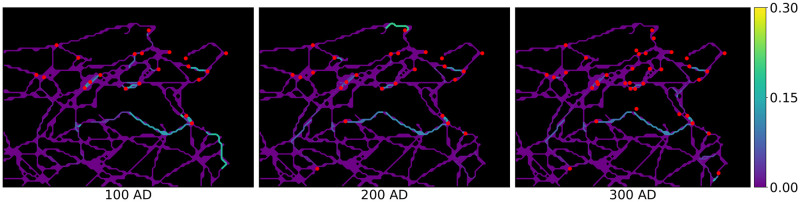
Temporal mean squared error between $\tilde{\alpha }\left(e\right)$ and *α*(*e*). Romanised municipalities are marked in red. Roman roads network lines reproduced with permission from Ancient World Mapping Center [61] under a CC BY licence, original copyright 2015.

In Fig 10, we plot this error for every time frame from averaging over 1000 simulations. We observe that the sensitivity to perturbations is much stronger on the road segments in areas with less municipalities, i.e. in the remote regions (hinterland). The affected areas are different for individual time frames and they mainly depend on the Romanisation times of municipalities in the less dense areas.

### 4.4 Stability analysis

In this section, we will test how vulnerable our model is to missing data. The motivation for this is the possibility that there existed Romanised municipalities in the region we are considering which do not occur in our data. Hence, the results of a stable model should not greatly differ when excluding one or multiple municipalities from the model. To evaluate the stability of our model, we select a municipality *c* and delete it from the data obtaining the altered data $D^{\left(c\right)}$. We compare the resulting activation probabilities $\alpha_{e}^{\left(c\right)}\left(t\right)$ of the road segments to the activation probability *α*_*e*_(*t*) of the unaltered data. Then, we consider the squared distance between the two activation probabilities at each point in time

$$\begin{array}{c} v_{e}^{\left(c\right)}\left(t\right)={\left(\alpha_{e}^{\left(c\right)}\left(t\right)-\alpha_{e}\left(t\right)\right)}^{2}. \end{array}$$ (12)

**Fig 11:**
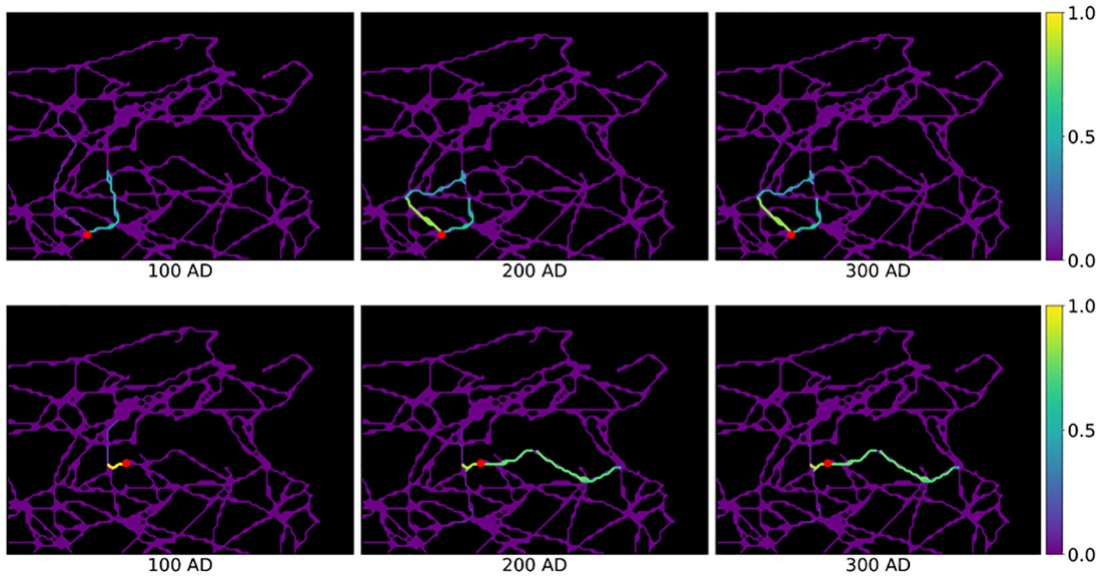
Temporal squared distance $v_{e}^{\left(c\right)}\left(t\right)$ when excluding the city of Cillium (top) and respectively Mactaris (bottom) from the model. The city excluded is marked in red. Roman roads network lines reproduced with permission from Ancient World Mapping Center [60] under a CC BY licence, original copyright 2015.

Evaluating this quantity over all choices of *c*, we observe that deleting municipalities has only local effects, i.e. only the activation probabilities of road segments that are close to *c* are altered. Furthermore, this effect is more pronounced in the areas with less municipalities, i.e. in the remote regions. In Fig 11, we show $v_{e}^{\left(c\right)}\left(t\right)$ for *t* = 100, 200, 300 AD and for a choice of two cities, Cillium and Mactaris, which have low stability.

## 5 Conclusions and outlook

In this paper, we studied the interplay between the cultural phenomena of Romanisation spreading and the Roman infrastructure evolving in ancient Tunisia. Since the various archaeological, historical and philological sources on such complex interactions cannot always be in agreement [79, 94], formal and probabilistic approaches serve to integrate seemingly contradictory evidence into a coherent model.

Building on selected concepts of epidemiological modelling, we introduced a new mathematical model and its spatio-temporal concept of Roman road activation. By integrating available archaeological records on the Roman settlement system, we showed how this model can be used to explore the potential spatio-temporal diffusion pattern of Romanisation as visible in our study region’s settlement system. We also offered an interpretation of how these formal results might relate to the known historical record of ancient Tunisia under Roman rule.

Beyond the scope of our case study on the Romanisation of North Africa, we further intended to make a contribution to the generic mathematical toolkit available for studying complex social diffusion. The research and model design were driven by conscious decisions as much as by the nature of the archaeological body of evidence. It was primarily the latter which defined the limits of analytical flexibility and led to a pragmatic model design with a focus on the Roman road network and selected attributes of the settlement system.

Thus, apart from the specific model outcomes, we also wanted to demonstrate more generally, that:

- Any seemingly intractably complex cultural phenomenon can be broken down into analytical components of manageable complexity, that still provide useful insights.
- Some methods and concepts from the domain of epidemiology can be transferred into that of archaeology in an explicit and meaningful way, without going to the extreme of considering cultural diffusion a form of infectious disease.
- In the age of Big Data and artificial intelligence, there is still an important place for plain mathematical models that can deal elegantly and transparently with typical archaeological data.

Despite (or perhaps because of) the richness of the archaeological record, reconstructing the actual temporal sequence and spatial spread of the Roman transformation of northern Africa is no simple task. Much uncertainty stems from the fact that neither the surviving written records nor the other archaeological evidence contain sufficiently dense and accurate temporal information (one of the obvious weaknesses of our approach is the fact that we used a largely reconstructed and only partially observed roads network).

In our case, the method for filling the temporal gaps is the use of a mathematical model that will produce a plausible temporal sequence in which individual segments of the ancient road network might have been activated during the course of the Roman reshaping of the existing settlement system. In this way, a spatio-temporally explicit picture of a core aspect of Romanisation in ancient Tunisia was inferred.

Some intricacies of the analysis do not arise from the spatial structure of the data as such, but rather from the fact that the preserved and observable remains of the road network represent an agglomeration of regional sub-networks [95], each of them potentially shaped by different optimisation criteria. Like any modern traffic network, the Roman roads served different purposes and priorities, and were supported by different funding and maintenance models. Roman infrastructure tends to overlay and integrate previously existing roads, trade routes and settlements. It also tends to augment existing structures with special-purpose elements, such as military roads, and new systems of land division for agricultural use.

Due to such sources of uncertainty, a model like ours is exploratory in general and inferential under the condition of strictly controlled (known) parameters. It is only as good as the data and results depend, among other things, on initial conditions. It is, however, at the same time capable of making (selected aspects of) a complex phenomenon explicit and disentangling layers of information.

Any mathematical model can only represent selected aspects of reality and will always be a compromise between explanatory power and demands on data quality. We make our data, both the subset used in this paper, as well as the complete collection, openly available, in the hope that researchers will use it to experiment, gain new insights and improve upon our work.

## Supporting information

S1 Appendix (ZIP)

## References

[1] Hingley R. Globalizing Roman Culture. Unity, Diversity and Empire. Routledge; 2005.

[2] Geraghty RM. The Impact of Globalization in the Roman Empire 200 BC–AD 100. The Journal of Economic History. 2007;67(4):1036–1061. doi: 10.1017/S0022050707000484

[3] Pitts M, Versluys MJ, editors. Globalisation the Roman World: World History, Connectivity and Material Culture. Cambridge University Press; 2014.

[4] Morley N. The Roman Empire. Roots of Imperialism. Pluto Press; 2015.

[5] Adkins L, Adkins RA. Handbook to Life in Ancient Rome. Oxford University Press; 1998.

[6] Woolf G. Becoming Roman. The Origins of Provincial Civilization in Gaul. Cambridge University Press; 1998.

[7] Schörner G, editor. Romanisierung—Romanisation: Theoretische Modelle und praktische Fallbeispiele. BAR Publishing; 2005.

[8] Brughmans T, Wilson A. Simulating Roman Economies: Theories, Methods, and Computational Models. Oxford University Press; 2022.

[9] Hingley R. Romanization. In: Smith C, editor. Encyclopedia of Global Archaeology. Springer; 2014. p. 6373–6380.

[10] Hoyos D. The Carthaginians. Routledge; 2010.

[11] Baronowski DW. Polybius on the Causes of the Third Punic War. Classical Philology. 1995;90(1):16–31. doi: 10.1086/367442

[12] Ros KE. The Roman Theater at Carthage. American Journal of Archaeology. 1996;100(3):449–489. doi: 10.2307/507025

[13] Mattingly DJ, Hitchner RB. Roman Africa: An Archaeological Review. The Journal of Roman Studies. 1995;85:165–213. doi: 10.2307/301062

[14] Oltean IA. Dacia. Landscape, Colonization and Romanization. Routledge; 2007.

[15] Ardeleanu S. Numidia Romana? No. 38 in Archäologische Forschungen. Reichert Verlag; 2021. Available from: https://zenon.dainst.org/Record/002025442.

[16] Alcock SE, Egri M, Frakes JFD. Beyond Boundaries: Connecting Visual Cultures in the Provinces of Ancient Rome. Getty Publications—Series. Getty Publications; 2016. Available from: https://books.google.de/books?id=AOlYDQAAQBAJ.

[17] Feldman LH. Rabbinic insights on the decline and forthcoming fall of the Roman empire. Journal for the Study of Judaism in the Persian, Hellenistic, and Roman Period. 2000;31(3):275–297. doi: 10.1163/157006300X00134

[18] Dyson SL. Native Revolt Patterns in the Roman Empire. In: Temporini H, editor. Politische Geschichte (Provinzen und Randvölker: Allgemeines Britannien, Hispanien, Gallien). vol. 3 of Austieg und Niedergang der römischen Welt (ANRW)/Rise and Decline of the Roman World. De Gruyter; 1975. p. 138–175.

[19] Bispham E. Roman Europe. In: Blanning TCW, editor. The Short Oxford History of Europe. Oxford University Press; 2008.

[20] Appelbaum A. Hidden Transcripts in King-Parables: Windows on Rabbinic Resistance to Rome. Jewish Studies Quarterly. 2012;17(4):287–301. doi: 10.1628/094457010793468715

[21] Ben Zeev M. New Insights into Roman Policy in Judea on the Eve of the Bar Kokhba Revolt. Journal for the Study of Judaism in the Persian, Hellenistic, and Roman Period. 2018;49(1):84–107. doi: 10.1163/15700631-12481193

[22] Ben Abed A. Tunisian Mosaics. Treasures from Roman Africa. Getty Press; 2006.

[23] Palmieri L. Romanization and definition of commercial areas in Africa Proconsularis: the examples of Leptis Magna and Thugga. In: Corsi C, Vermeulen F, editors. Changing Landscapes. The Impact of Roman Towns in the Western Mediterranean. Proceedings of the International Colloquium, Castelo de Vide—Marvão 15th–17th May 2008. Ante QUem; 2010. p. 385–392.

[24] Scheding P. Chapter 11: Micro-regional Urbanism: An Ancient Urban Landscape in Roman North Africa. In: de Ligt L, Bintliff J, editors. Regional Urban Systems in the Roman World, 150 BCE–250 CE. Leiden, The Netherlands: Brill; 2020. p. 350–374. Available from: https://brill.com/view/book/edcoll/9789004414365/BP000009.xml.

[25] Laurence R. The roads of Roman Italy: Mobility and cultural change. Routledge; 1999.

[26] Kissel T. Lokale Identität und imperiale Herrschaft. Römische Straßen in Arabien als Wegbereiter von Akkulturationsprozessen. In: Sprache und Kultur in der kaiserlichen Provinz Arabia. Althistorische Beiträge zur Erforschung von Akkulturationsphänomenen im römischen Nahen Osten. No. 4 in Mainzer Althistorische Studien. Scripta Mercaturae; 2003. p. 12–69.

[27] Cioffi RL. Travel in the Roman World. In: Oxford Handbook Topics in Classical Studies. Oxford University Press; 2016.

[28] Flückiger M, Hornung E, Larch M, Ludwig M, Mees A. Roman Transport Network Connectivity and Economic Integration. The Review of Economic Studies. 2021-07;89(2):774–810. 10.1093/restud/rdab036

[29] Filiasi G. Delle Strade Romane che Passavano Anticamente pel Mantovano (reprint 1985). Arnaldo Forni Editore; 1792.

[30] Kolb A, editor. Roman Roads. New Evidence—New Perspectives. Berlin, Boston: De Gruyter; 2019.

[31] Carreras C, De Soto P. The Roman transport network: a precedent for the integration of the European mobility. Historical Methods: A Journal of Quantitative and Interdisciplinary History. 2013;46(3):117–133. doi: 10.1080/01615440.2013.803403

[32] Verhagen P, Nuninger L, Groenhuijzen MR. Modelling of pathways and movement networks in archaeology: an overview of current approaches. Finding the limits of the limes: Modelling demography, economy and transport on the edge of the Roman empire. 2019; p. 217–249. doi: 10.1007/978-3-030-04576-0_11

[33] Groenhuijzen MR, Verhagen P. Testing the robustness of local network metrics in research on archeological local transport networks. Frontiers in digital humanities. 2016;3:6. doi: 10.3389/fdigh.2016.00006

[34] Prignano L, Font-Pomarol L, Morer I, Lozano S. Infrastructures connecting people: A mechanistic model for terrestrial transportation networks. Environment and Planning B: Urban Analytics and City Science. 2023;50(8):2254–2272. 10.1177/23998083231174024

[35] Cook RM, Woodhead AG. The Diffusion of the Greek Alphabet. American Journal of Archaeology. 1959;63(2):175–178. doi: 10.2307/501755

[36] Edmonson MS. Neolithic Diffusion Rates. Current Anthropology. 1961;2(2):71–102. doi: 10.1086/200169

[37] Davis DD. Investigating the Diffusion of Stylistic Innovations. Advances in Archaeological Method and Theory. 1983;6:53–89. doi: 10.1016/B978-0-12-003106-1.50007-9

[38] Henrich J. Cultural Transmission and the Diffusion of Innovations: Adoption Dynamics Indicate That Biased Cultural Transmission Is the Predominate Force in Behavioral Change. American Anthropologist. 2001;103(4):992–1013. doi: 10.1525/aa.2001.103.4.992

[39] Wahl F. Does European development have Roman roots? Evidence from the German Limes. Journal of Economic Growth. 2017-07;22(3):313–349. doi: 10.1007/s10887-017-9144-0

[40] Licio V. When History Leaves a Mark: A New Measure of Roman Roads. Italian Economic Journal. 2020;7(1):1–35. doi: 10.1007/s40797-020-00120-5

[41] Gleason JP. Roman roads in Gaul: how lines of communication and basing support operational reach. SAMS Monograph. School of Advanced Military Studies; 2013.

[42] Coquillette DR. 1. In: The Glory that was Rome. 2nd ed. Carolina Academic Press; 2004. p. 1–35.

[43] Woolf G. Imperialism, Empire and the Integration of the Roman Economy. World Archaeology. 1992;23(3):283–293. doi: 10.1080/00438243.1992.9980180

[44] Temin P. The Economy of the Early Roman Empire. The Journal of Economic Perspectives. 2006;20(1):133–151. doi: 10.1257/089533006776526148

[45] Frank T. The Inscriptions of the Imperial Domains of Africa. The American Journal of Philology. 1926;47(1):55–73. doi: 10.2307/289848

[46] Broughton TRS. The romanization of Africa proconsularis. John Hopkins Press; 1929.

[47] Duncan-Jones RP. Wealth and Munificence in Roman Africa. Papers of the British School at Rome. 1963;31:159–177. doi: 10.1017/S0068246200001690

[48] Hitchner RB. Olive Production and the Roman Economy: The Case for Intensive Growth. In: Amouretti MC, Brun JP, editors. La production du vin et de l’huile en Méditerranée. No. 26 in Bulletin de Correspondance Héllenique Suppl.. Ecole française d’Athènes; 1993. p. 499–503.

[49] Mattingly DJ. Oil for export? A Oil for Export? A comparison of Libyan, Spanish and Tunisian olive oil production in the Roman Empire. Journal of Roman Archaeology. 1988;1:33–56. doi: 10.1017/S1047759400009971

[50] Aomar A. L’Africa romana: mobilitÃdelle persone e dei popoli, dinamiche migratorie, emigrazioni ed immigrazioni nelle province occidentali dell’Impero romano. In: Akerraz A, Ruggeri P, Siraj A, Vismara C, editors. Atti del XVI convegno di studio, Rabat, 15-19 dicembre 2004. Carocci; 2006. p. 1396–2108.

[51] Mattingly DJ. A new study of olive oil (and wine?) production in northern Tunisia. Journal of Roman Archaeology. 2009;22(2):715–720. doi: 10.1017/S1047759400021371

[52] Kehoe D. Lease Regulations for Imperial Estates in North Africa: Part I. Zeitschrift für Papyrologie und Epigraphik. 1984;56:193–219.

[53] Johannesen R. The Textile Industry in Roman Africa. The Classical Journal. 1954;49(4):157–160.

[54] Peacock DPS, Bejaoui F, Ben Lazreg N. Roman pottery production in central Tunisia. Journal of Roman Archaeology. 1990;3:59–84. doi: 10.1017/S1047759400010849

[55] Lazzarini L, Agus M, Cara S. The ancient quarries of the neri antichi (black limestones) from Zeugitania (Tunisia). Marmora. 2007;2:59–70.

[56] Isern N, Zilhão J, Fort J, Ammerman AJ. Modeling the role of voyaging in the coastal spread of the Early Neolithic in the West Mediterranean. Proceedings of the National Academy of Sciences. 2017;114(5):897–902. doi: 10.1073/pnas.1613413114 28096413 PMC5293084

[57] McLean A, Rubio-Campillo X. Beyond Least Cost Paths: Circuit theory, maritime mobility and patterns of urbanism in the Roman Adriatic. Journal of Archaeological Science. 2022;138:105534. doi: 10.1016/j.jas.2021.105534

[58] Hitchner RB. Roads, Integration, Connectivity, and Economic Performance in the Roman Empire. In: Alcock SE, Bodel J, Talbert RJA, editors. Highways, Byways, and Road Systems in the Pre-Modern World. Wiley; 2012. p. 222–234.

[59] Natural Earth;. Available from: http://www.naturalearthdata.com.

[60] The Ancient World Mapping Center;. Available from: http://awmc.unc.edu.

[61] Hobson M. Chapter 9: Roman Towns and the Settlement Hierarchy of Ancient North Africa: A Bird’s-Eye View. In: de Ligt L, Bintliff J, editors. Regional Urban Systems in the Roman World, 150 BCE–250 CE. Leiden, The Netherlands: Brill; 2020. p. 281–323. Available from: https://brill.com/view/book/edcoll/9789004414365/BP000009.xml.

[62] Scheding P. Urbaner Ballungsraum im römischen Nordafrika: zum Einfluss von mikroregionalen Wirtschafts- und Sozialstrukturen auf den Städtebau in der Africa Proconsularis. vol. 16. Wiesbaden: Dr. Ludwig Reichert Verlag; 2019.

[63] Hitchner RB. The Organization of Rural Settlement in the Cillium-Thelepte Region (Kasserine, Central Tunisia). L’Africa Romana. 1989;6:387–402.

[64] Babelon E, Cagnat R, Reinach S. Atlas archéologique de la Tunisie: édition spéciale des cartes topographiques. E. Leroux; 1893.

[65] Wilson. Urban Production in the Roman World: The View from North Africa. Papers of the British School at Rome. 2002;70:231–273. doi: 10.1017/S0068246200002166

[66] Kehoe D. Lease Regulations for Imperial Estates in North Africa: Part II. Zeitschrift für Papyrologie und Epigraphik. 1985;56:151–172.

[67] Cappelletto E. Urbanization in Africa Proconsularis in the Time of Emperor Claudius. In: Bombardieri L, D’Agostino A, Guarducci G, Orsi V, Valentini S, editors. Identity and Connectivity. Proceedings of the 16th Symposium on Mediterranean Archaeology, Florence, Italy, 1–3 March 2012. vol. I of BAR International Series. BAR Publishing; 2013. p. 613–622.

[68] Kandler A, Perreault C, Steele J. Editorial—Cultural evolution in spatially structured populations: A review of alternative modeling frameworks. Advances in Complex Systems. 2012;15(01n02):(online). doi: 10.1142/S0219525912030014

[69] Scholnick JB. The spatial and temporal diffusion of stylistic innovations in material cultural. Advances in Complex Systems. 2012;15:(online). doi: 10.1142/S0219525911003244

[70] Silva F, Steele J. Modeling boundaries between converging fronts in prehistory. Advances in Complex Systems. 2012;15(01n02):(online). doi: 10.1142/S0219525911003293

[71] Margary ID. Roman roads in Britain. John Baker; 1973.

[72] Chevallier R. Roman roads (translated by N. H. Field). University of California Press; 1976.

[73] Herzog I, Schröer S. Reconstruction of Roman Roads and Boundaries in Southern Germany. In: Proceedings of the 22nd International Conference on Cultural Heritage and New Technologies held in Vienna, Austria November 2017; 2017.

[74] Bishop MC. The secret history of Roman roads in Britain. Pen and Sword; 2020.

[75] Salama P. Les voies romaines de l’Afrique du Nord. Gouvernement général de l’Algérie. Impr. officielle; 1951.

[76] Barbery J, Delhoume JP. La voie romaine de piedmont Sufetula-Masclianae (Djebel Mrhila, Tunisie centrale). Antiquités Africaines. 1982;18:27–43. doi: 10.3406/antaf.1982.1083

[77] de Vos Raaijmakers M. Twin Roads: the Road Carthage-Theveste and the via nova Rusicadensis; some Observations and Questions. In: Roman Roads. De Gruyter; 2019. p. 338–374.

[78] Kissel T. In: Road-building as a munus publicum. BRILL; 2002-01. p. 127–160.

[79] Stone DL. Africa in the Roman Empire: Connectivity, the Economy, and Artificial Port Structures. American Journal of Archaeology. 2014;118(4):565–600. doi: 10.3764/aja.118.4.0565

[80] Wilmanns G. Inscriptiones Africae latinae. vol. 3 of Corpus inscriptionum latinarum. Berolini: G. Reimerum; 1881.

[81] Ellis L, Kidner FL. Milestones, Communication, and Political Stability. Ashgate; 2004.

[82] Rathmann M. Untersuchungen zu den Reichsstraßen in den westlichen Provinzen des Imperium Romanum. Bonner Jahrbücher des Rheinischen Landesmuseums in Bonn und des Vereins von Altertumsfreunden im Rheinlande: Beiheft; 55. Mainz: von Zabern; 2003.

[83] Ducke B, Schweigart F, Chemnitz R. Archaeological and spatial data on the Romanisation of Northern Africa (146 BC to c. 400 AD); 2022. Available from: https://www.zib.de/tes-data-sets/ds-romanization.

[84] Talbert JA. Barrington Atlas of the Greek and Roman World; 2000.

[85] McCormick M, Huang G, Zambotti G, Lavash J. Roman Road Network (version 2008); 2013.

[86] Kermack WO, McKendrick AG. A contribution to the mathematical theory of epidemics. Proceedings of the royal society of london Series A, Containing papers of a mathematical and physical character. 1927;115(772):700–721.

[87] Gomez-Rodriguez M, Leskovec J, Krause A. Inferring networks of diffusion and influence. ACM Transactions on Knowledge Discovery from Data (TKDD). 2012;5(4):1–37. doi: 10.1145/2086737.2086741

[88] Kempe D, Kleinberg J, Tardos E. Maximizing the Spread of Influence through a Social Network. In: Proceedings of the Ninth ACM SIGKDD International Conference on Knowledge Discovery and Data Mining. KDD’03. New York, NY, USA: Association for Computing Machinery; 2003. p. 137–146. Available from: 10.1145/956750.956769.

[89] van Seventer JM, Hochberg NS. Principles of Infectious Diseases: Transmission, Diagnosis, Prevention, and Control. International Encyclopedia of Public Health. 2017; p. 22–39. doi: 10.1016/B978-0-12-803678-5.00516-6

[90] Dirnberger M, Kehl T, Neumann A. NEFI: Network Extraction From Images. Scientific Reports. 2015;5(1):15669. doi: 10.1038/srep15669 26521675 PMC4629128

[91] Baptista D, De Bacco C. Principled network extraction from images. Royal Society Open Science. 2021;8(7):210025. doi: 10.1098/rsos.210025 34350013 PMC8316801

[92] Dijkstra EW. A note on two problems in connexion with graphs. Numerische Mathematik. 1959;1(1):269–271. doi: 10.1007/BF01386390

[93] Djurdjevac Conrad N, Chemnitz R, Kostre M, Schweigert F, Fless F, Schuette C, et al. Implementation of the methods from the paper “A Mathematical perspective on Romanisation: Modelling the Roman road activation process in ancient Tunisia”; 2024. Available from: 10.5281/zenodo.11234841. PMC11424008 39321114

[94] Brughmans T, Hanson J, Mandich M, Romanowska I, Rubio-Campillo X, Carrignon S, et al. Formal modelling approaches to complexity science in Roman studies: a manifesto. Theoretical Roman Archaeology Journal. 2019;2(1):1–19. doi: 10.16995/traj.367

[95] Kostre M, Sunkara V, Schütte C, Conrad ND. Understanding the Romanization Spreading on Historical Interregional Networks in Northern Tunisia. Applied Network Science. 2022;7. doi: 10.1007/s41109-022-00492-w

