# Supplementary material for: A mathematical perspective on Romanisation: Modelling the Roman road activation process in ancient Tunisia
Source: PLoS One. 2024 Sep 25;19(9):e0309752. doi: 10.1371/journal.pone.0309752 (PMC11424008; doi:10.1371/journal.pone.0309752)
Supplement: S1 Appendix — (ZIP) [file pone.0309752.s001.zip › S1_Appendix.pdf]

1 Further details on data compiled for the study

We compiled freely available (under Creative Commons licenses or as part of the public domain) data from a number of sources: `https://www.naturalearthdata.com` (topographic background data from the Natural Earth project: [1]), `http://awmc.unc.edu` (historical map data from the Ancient World Mapping Center), `https://idai.world` (the iDAI.world online data services: [2]), `https://imperium.ahlfeldt.se` (the Digital Atlas of the Roman Empire), `https://pleiades.stoa.org` (a gazetteer of ancient places: [3]), `https://edh.ub.uni-heidelberg.de` (Epigraphik-Datenbank Clauss/Slaby: [4]), and `https://edh.ub.uni-heidelberg.de` (Epigraphic Database Heidelberg: [5]). In addition, we consulted [6] in its printed form, as well as `https://www.trismegistos.org`, a proprietary online database ([7]).

The data compiled for our study is available in its entirety ('main'), in a standardised and cleaned version ('trunk') and as the exact subset ('branch') that was used to build the model discussed here. It was first released in a single package as part of the MATH+ Thematic Einstein Semester on the 'The Mathematics of Complex Social Systems: Past, Present, and Future' in 2022 ([8]).

Our main dataset contains c. 6800 archaeological observations (sites) that pertain to architectural, structural and administrative features of the remains of Roman era settlements within the territorial boundaries of modern day Tunisia. Some sites in the main dataset are outliers in the territories of neighbouring modern nations, and some could not be assigned geographic coordinates at all (places associated with finds by name, but never located with certainty).

Most sites are close to or within the boundaries of modern municipalities and are thus primarily referred to by their modern names. Arabic place names have been transcribed to the Latin alphabet using French transliterations (but with accent characters removed so that pure ASCII encoding can be used). The transcriptions are, however, not fully consistent. Latin names were preserved in the data where known.

In this context, it is crucial to note that the concept of an exactly located and delineated site (marked by a single point on the map) is an analytical simplification of the real nature of the archaeological record. The latter occurs as a distribution of surface finds scattered across the landscape. This aspect is encoded in the data by using spatial qualifiers such as 'near' or 'between'.

Each site record is also enriched with categorised observations (in the form of a binary presence/absence encoding) on the presence of features that are commonly associated with the rule and activities of the Roman Empire.

The trunk dataset represents a post-processed version of the main archive that has been quality-checked, simplified and standardised to a degree which makes it immediately suitable for further processing and analysis in any software that can ingest tabular ASCII data. The trunk dataset is only slightly smaller than the main one, with outliers, duplicates and sites with unknown locations removed. Standardisation was followed by normalisation, i.e. splitting the data into several tables (linked via a unified primary key field), such that there are no redundancies in the records. Despite all of this, the trunk dataset preserves the richness of the original (main) version.

Further details can be found in the README files included in the dataset distribution.

2 Pseudocode for obtaining the activation probabilities

Data: influences $\pi(c, c')$, paths $\Gamma_{c,c'}$, times of Romanisation $t_{rom}(c)$, set of edges $\mathcal{E}$, set of municipalities $\mathcal{C}$, maximum time T
Result: activation probability $\alpha_e(t)$

Initialise: $P_c = \emptyset$;
$\phi_c(e) = 0$;
$\beta_e(t) = 1$;

for $c \in \mathcal{C}$ **do**
  ⟨ Determine the set of parents P_c and their probabilities $\pi(p, c)$ ⟩
  $\pi_{sum} = 0$;
  for $p \in \mathcal{C}$ **do**
    if $t_{rom}(p) < t_{rom}(c)$ **then**
      P_c.add(p);
      $\pi_{sum} = \pi_{sum} + \pi(p, c)$;
    end
  end

  ⟨ Determine the probability $\phi_c(e)$ of e of activation by c ⟩
  for $p \in P_c$ **do**
    for $e \in \Gamma_{p,c}$ **do**
      $\phi_c(e) = \phi_c(e) + \frac{\pi(p,c)}{\pi_{sum}}$;
    end
  end

  ⟨ Update the probabilities $\beta_e(t)$ in the respective time frame s ⟩
  for $e \in \mathcal{E}$ **do**
    for $t_{rom}(c) \leq t \leq T$ **do**
      $\beta_e(t) = \beta_e(t) \cdot (1 - \phi_c(e))$;
    end
  end
end

$\alpha_e(t) = 1 - \beta_e(t)$;

Algorithm 1: Algorithm to compute activation probability of a road segment e at time t.

References

1. Vaughn Kelso N, Patterson T. Natural Earth Vector. Cartographic Perspectives. 2009;64:45–50.

2. Senst H, Riebschläger F, Watson J. iDAI.world: Die vernetzte Forschungsdatenplattform des Deutschen Archäologischen Instituts. obib Das offene Bibliotheksjournal. 2022;9(4). doi:10.5282/o-bib/5844.

3. Barker E, Simon R, Isaksen L, de Soto CaÃ±amares P. The Pleiades Gazetteer and the Pelagios Project. In: Berman ML, Mostern R, Southall H, editors. Enriching and Integrating Gazetteers. Bloomington: Indiana University Press; 2016. p. 97–109.

4. Clauss M. Epigraphik-Datenbank Clauss-Slaby (EDCS). Revue Archéologique de Narbonnaise. 2010;43:16–18.

5. Witschel C. Die Epigraphische Datenbank Heidelberg (EDH). In: Sellin V, Wollgast E, Zwies S, editors. Die Forschungsvorhaben der Heidelberger Akademie der Wissenschaften 1909-2009. 100 Jahre Heidelberger Akademie der Wissenschaften. Heidelberg: Universitätsverlag Winter; 2010. p. 227–232.

6. Stillwell R, MacDonald WL, McAlister MH. The Princeton Encyclopedia of Classical Sites. Princetion, N.J.: Princetion University Press; 1976.

7. Depauw M, Gheldof T. Trismegistos: An Interdisciplinary Platform for Ancient World Texts and Related Information. In: Bolikowski Ł, Casarosa V, Goodale P, Houssos N, Manghi P, Schirrwagen J, editors. Theory and Practice of Digital Libraries – TPDL 2013 Selected Workshops. Cham: Springer International Publishing; 2014. p. 40–52.

8. Ducke B, Schweigart F, Chemnitz R. Archaeological and spatial data on the Romanisation of Northern Africa (146 BC to c. 400 AD); 2022. Available from: https://www.zib.de/tes-data-sets/ds-romanization.
